# Modification of xylan in secondary walls alters cell wall biosynthesis and wood formation programs and improves saccharification

**DOI:** 10.1111/pbi.14487

**Published:** 2024-10-22

**Authors:** Pramod Sivan, János Urbancsok, Evgeniy N. Donev, Marta Derba‐Maceluch, Félix R. Barbut, Zakiya Yassin, Madhavi L. Gandla, Madhusree Mitra, Saara E. Heinonen, Jan Šimura, Kateřina Cermanová, Michal Karady, Gerhard Scheepers, Leif J. Jönsson, Emma R. Master, Francisco Vilaplana, Ewa J. Mellerowicz

**Affiliations:** ^1^ Umeå Plant Science Centre, Department of Forest Genetics and Plant Physiology Swedish University of Agricultural Sciences Umeå Sweden; ^2^ Division of Glycoscience, Department of Chemistry KTH Royal Institute of Technology, AlbaNova University Centre Stockholm Sweden; ^3^ RISE Research Institutes of Sweden Stockholm Sweden; ^4^ Department of Chemistry Umeå University Umeå Sweden; ^5^ Wallenberg Wood Science Centre (WWSC) KTH Royal Institute of Technology Stockholm Sweden; ^6^ Laboratory of Growth Regulators, The Czech Academy of Sciences & Faculty of Science Institute of Experimental Botany, Palacký University Olomouc Czechia; ^7^ Department of Chemical Engineering and Applied Chemistry University of Toronto Toronto Ontario Canada

**Keywords:** Glucuronoxylan, fungal xylanases, transgenic aspen, wood development, lignocellulose, secondary cell wall

## Abstract

Wood of broad‐leaf tree species is a valued source of renewable biomass for biorefinery and a target for genetic improvement efforts to reduce its recalcitrance. Glucuronoxylan (GX) plays a key role in recalcitrance through its interactions with cellulose and lignin. To reduce recalcitrance, we modified wood GX by expressing GH10 and GH11 endoxylanases from *Aspergillus nidulans* in hybrid aspen (*Populus tremula* L. × *tremuloides* Michx.) and targeting the enzymes to cell wall. The xylanases reduced tree height, modified cambial activity by increasing phloem and reducing xylem production, and reduced secondary wall deposition. Xylan molecular weight was decreased, and the spacing between acetyl and MeGlcA side chains was reduced in transgenic lines. The transgenic trees produced hypolignified xylem having thin secondary walls and deformed vessels. Glucose yields of enzymatic saccharification without pretreatment almost doubled indicating decreased recalcitrance. The transcriptomics, hormonomics and metabolomics data provided evidence for activation of cytokinin and ethylene signalling pathways, decrease in ABA levels, transcriptional suppression of lignification and a subset of secondary wall biosynthetic program, including xylan glucuronidation and acetylation machinery. Several candidate genes for perception of impairment in xylan integrity were detected. These candidates could provide a new target for uncoupling negative growth effects from reduced recalcitrance. In conclusion, our study supports the hypothesis that xylan modification generates intrinsic signals and evokes novel pathways regulating tree growth and secondary wall biosynthesis.

## Abstract 2

Fungal xylanases expressed in aspen decreased lignin content in the wood of transgenic lines due to downregulation of a subset of lignin biosynthetic genes. This greatly increased saccharification potential. Transgenic plants exhibited altered growth and several signaling and regulatory genes were found affected that could mediate these effects via xylobiose signaling.

## Introduction

Plant cell wall is a highly dynamic and heterogenous structure made by complex chemical organization of cellulose, diverse matrix polysaccharides, structural proteins and polyphenols (Albersheim *et al*., 2010). The structure, composition and molecular interaction of matrix polysaccharides determine cell shape and tensile properties necessary for the mechanical strength of cell wall. Xyloglucans and pectins form the matrix of primary cell wall and their interactions with cellulose microfibrils within highly hydrated architecture facilitate cell expansion. The secondary walls (SWs) are deposited in xylem cells after cell expansion and have a denser and thicker network of cellulose microfibrils with a SW‐specific combination of matrix polysaccharides including glucuronoxylan (GX) and glucomannan. Deposition of this SW polysaccharide network and its subsequent lignification starting from the primary wall makes further cell expansion impossible but provides xylem cells with mechanical strength and rigidity. The primary and secondary walls constitute wood biomass which is the most abundant renewable resource on Earth for sustainable production of eco‐friendly materials, chemicals and energy carriers (Bar‐On *et al*., 2018; Keegan *et al*., 2013; Martínez‐Abad *et al*., 2018).

Biosynthesis of cell wall components has been largely investigated by studying cell wall mutants like *murus* (*mur)* (Mertz *et al*., 2012), *irregular xylem* (*irx*) (Turner and Somerville, 1997), *fragile stem* (*fra*) (Zhong *et al*., 2005), *trichome birefringence‐like* (*tbl*) (Potikha and Delmer, 1995) and by systematic gene sequence analyses (Cantarel *et al*., 2009; Kumar *et al*., 2019). The enzymatic activities of several proteins have been characterized (e.g. Cavalier and Keegstra, 2006; Maris *et al*., 2011). However, it is still not well understood how the different activities are coordinated during cell wall biosynthesis to produce cell walls of required properties. Knowledge on the dynamic macromolecular changes during cell wall formation and modification in response to developmental and environmental changes is fundamental for our efforts to create plants with desired cell wall chemical composition suitable for industrial applications such as biorefinery and production of biomaterials (Pauly and Keegstra, 2008, 2010; Somerville and Bonetta, 2001).

The secondary cell wall of hardwood xylem contains approximately 25% (dry weight) of GX, which has been reported to have distinct structural features in terms of relative abundance of acetylation and glucuronidation. The distinct patterns of these decorations are organized in a major xylan domain that can adopt a two‐fold screw conformation compatible with the hydrophilic surface of cellulose, and a minor domain with a three‐fold screw conformation, which could interact with lignin (Bromley *et al*., 2013; Busse‐Wicher *et al*., 2014; Simmons *et al*., 2016; Yuan *et al*., 2016a, 2016b, 2016c; Grantham *et al*., 2017; Gupta *et al*., 2021). Thus, xylan molecules can differently affect cell wall architecture, and how their biosynthetic process is controlled to ensure formation of functional cell wall is not understood. This is complicated by the fact that, in addition to enzymatically driven xylan biosynthesis and modification, there are chemical catalysis‐driven processes that may occur in the cell wall changing its properties. For example, there is a long‐standing hypothesis that xylan provides nucleation sites for lignin polymerization, as suggested by the analysis of extracellular lignin after exogenously supplying xylan (Sapouna *et al*., 2023). In grasses, the ferulic acid linked to arabinose side chains of xylan is believed to initiate lignification (Hartley *et al*., 1990; Markwalder and Neukom, 1976; Ralph *et al*., 1995). In poplar, Ruel *et al*. (2006) proposed that hemicellulose‐lignin covalent linkage serves as an anchor for lignin polymerization.

Postsynthetic modification of the cell wall by overexpression of xylan‐modifying microbial enzymes represents a promising strategy to examine the contributions of different xylan structures to cell wall functions and to investigate mechanisms regulating cell wall biosynthesis in response to xylan integrity defects (Pogorelko *et al*., 2011). The cell wall modification by overexpression of xylan‐acting microbial enzymes has also been demonstrated to decrease biomass recalcitrance (Gandla *et al*., 2015; Pawar *et al*., 2016, 2017; Pogorelko *et al*., 2011; Pramod *et al*., 2021). Similarly, xylan structure defects caused by suppression of native xylan biosynthetic genes altered plant cell wall architecture and improved saccharification (Donev *et al*., 2018). These experiments demonstrated the potential use of plants compromised in xylan integrity for biorefinery applications, and in some cases revealed activation of biotic stress and growth responses triggered by xylan modification. However, their effects on cell wall developmental pathway received little or no attention.

In the present study, we report changes in xylem cell wall chemistry and resulting modifications in cell wall biosynthesis and xylem cell developmental programs in transgenic aspen overexpressing endo‐1,4‐β‐D‐xylanases of GH10 and GH11 families from *Aspergillus nidulans* in apoplast of developing xylem cells. Endo‐1,4‐β‐D‐xylanases (EC 3.2.1.8) cleave internal 1,4‐β‐xylosidic bonds in xylan backbones, producing low‐molecular‐weight (MW) heteroxylans and unsubstituted or branched xylo‐oligosaccharides (XOS) (Pollet *et al*., 2010; Reilly, 1981). The products of GH10 and GH11 xylanases slightly differ because only GH10 can accommodate a substituted xylosyl residue at the −1 subsite of the active site whereas both families require unsubstituted xylosyl residue at the +1 subsite (Biely *et al*., 1997; Kojima *et al*., 2022; Kolenová *et al*., 2006; Pell *et al*., 2004; Vardakou *et al*., 2008). The expression of xylanases altered xylem cell wall biosynthetic program and modified cambial activity suggesting the loss of xylan integrity in SWs is sensed by differentiating xylem cells. The resulting lignocellulosic biomass had substantially increased saccharification potential. However, the plants' growth was affected and uncoupling of the two effects is needed before such a strategy could be used for practical deployment.

## Results

### Microbial xylanases affected growth and vascular tissue differentiation pattern in aspen

**Figure 1:**
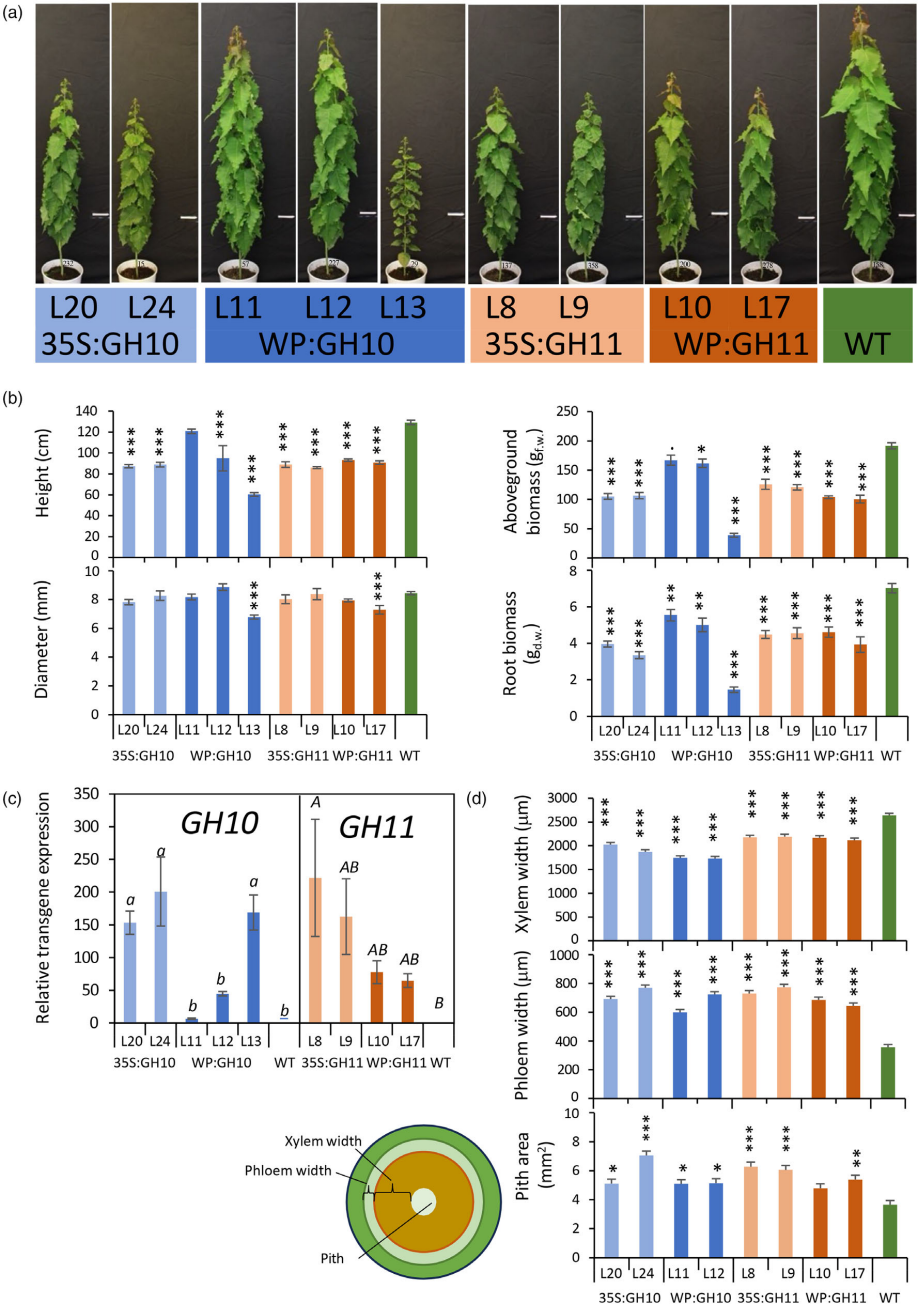
Growth of transgenic lines expressing GH10 and GH11 xylanases. (a) Morphology of 9‐week‐old plants. Bar =10 cm. (b) Plant size. (c) Expression of transgenes by RT‐qPCR normalized to the lowest‐expressing transgenic line. Different letters indicate significant difference among averages (*P* ≤ 0.05, Tukey test). (d) Morphometric data on stem anatomy as shown on the diagram, based on internode 40. Means ± SE; *N* = 6 trees for transgenic lines and 14 for WT in (b), *N* = 3 for transgenic lines and 6 for WT in (c), 2 trees × 10 radii or 2 trees × 2 sections in (d). ・*P* ≤ 0.1; *P ≤ 0.05; **P ≤ 0.01; ***P ≤ 0.001 for comparisons with WT by Dunnett's test.

To affect xylan in woody tissues, we expressed fungal GH10 and GH11 xylanases under control of either the 35S promoter for high ubiquitous expression or the wood‐specific promoter (WP) for more targeted expression in cells developing secondary walls (Ratke *et al*., 2015). The xylanases were directed to the apoplast by either fungal gene native signal peptide (in the case of *GH11*) or aspen gene signal peptide (in the case of *GH10*) as described and experimentally validated in the previous study (Barbut *et al*., 2024). The generated transgenic aspen lines showed clear morphological changes (Figure 1a). All growth parameters (stem height and diameter, aboveground and root biomass) were significantly affected compared to the wild‐type (WT) (Figure 1b). Transgene transcript levels were higher in 35S promoter lines than in WP lines (Figures 1c and S1), which did not correlate with growth penalty. However, in three WP:GH10 lines with different transgene levels, there was a clear negative impact of transgene transcript level on height and biomass production. Although radial growth was not affected in the majority of transgenic lines, the measurement of secondary vascular tissues from transverse sections revealed increased secondary phloem and decreased secondary xylem production (Figure 1d). The pith area of transgenic lines was also significantly increased. These observations indicate that xylanases stimulated growth of pith and had a major impact on cambial activity shifting it from xylem to phloem production.

**Figure 2:**
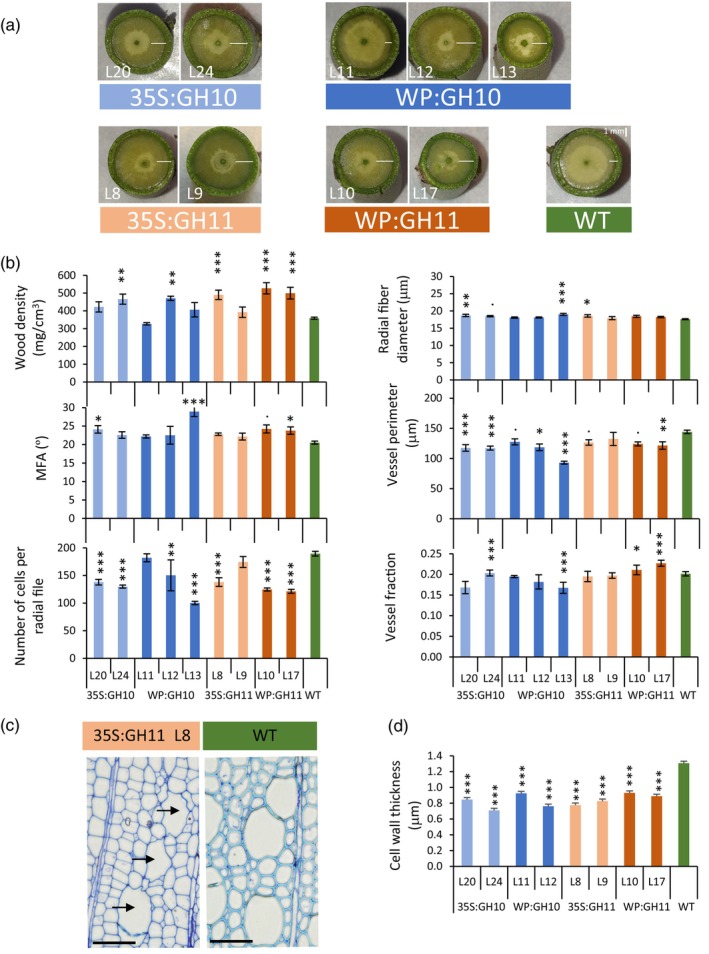
Wood quality traits of transgenic lines expressing GH10 and GH11 xylanases determined by SilviScan and anatomical analyses. (a) Appearance of SilviScan wood samples freshly dissected from the stems. Note the wider wet‐looking zone (white bars) in transgenic lines. (b) Different wood quality traits measured by SilviScan. Vessel fraction refers to the fraction of wood surface covered by vessels. (c) Typical appearance of wood in xylanase‐expressing plants. Note a reduction in cell wall thickness, *irregular xylem* phenotype (collapsed vessels, black arrows) and altered cell wall staining properties. Toluidine blue stained wood cross sections. Sections of other lines are shown in Figure S2. (d) Secondary wall thickness measured by transmission electron microscopy analysis. WT, wild type; MFA, cellulose microfibril angle. Data in b and d are means ± SE, *N* = 6 for transgenic lines and 24 for WT in b, or 2 trees × 3 images × 4 measurement in d. *P ≤ 0.05; **P ≤ 0.01; ***P ≤ 0.001 for comparisons with WT by Dunnett's test.

Intriguingly, the appearance of freshly cut stems of transgenic lines was altered. All lines, but the low‐expressing line WP:GH10_11, showed a markedly increased zone of wet xylem, which normally indicates developing and not fully lignified xylem (Figure 2a). The stems were also much easier to cut, suggesting changes in cell wall properties. Indeed, SilviScan analysis (Figure 2b) showed that several transgenic lines had higher wood density or increased cellulose microfibril angle (MFA). The number of xylem cells per radial file was reduced in transgenic lines confirming microscopy analyses. Furthermore, an increase in vessel fraction with concomitant decrease in vessel perimeter and an increase in fibre diameter were observed in several transgenic lines, suggesting that xylem cell fate and xylem cell expansion were also affected by the xylanases.

Analysis of semi‐thin transverse sections stained with toluidine blue O (TBO) revealed a substantial decrease in cell wall thickness and frequent occurrence of *irregular xylem* (*irx*) phenotype (Figures 2c,d and S2). Moreover, a shift in TBO colour from cyan in WT to violet‐blue in transgenic lines suggested a decrease in lignification. This was confirmed by analysis of lignin autofluorescence in the wood sections of transgenic and WT plants (Figure S3). Lignin autofluorescence images also revealed large wood areas in transgenic plants with very low signal, which possibly represent patches of tension wood (TW). All these changes were attenuated in the line WP:GH10_11 that had lower transgene expression compared to other lines.

### Xylanase expression had a major impact on the content and composition of wood matrix sugars and lignin

**Figure 3:**
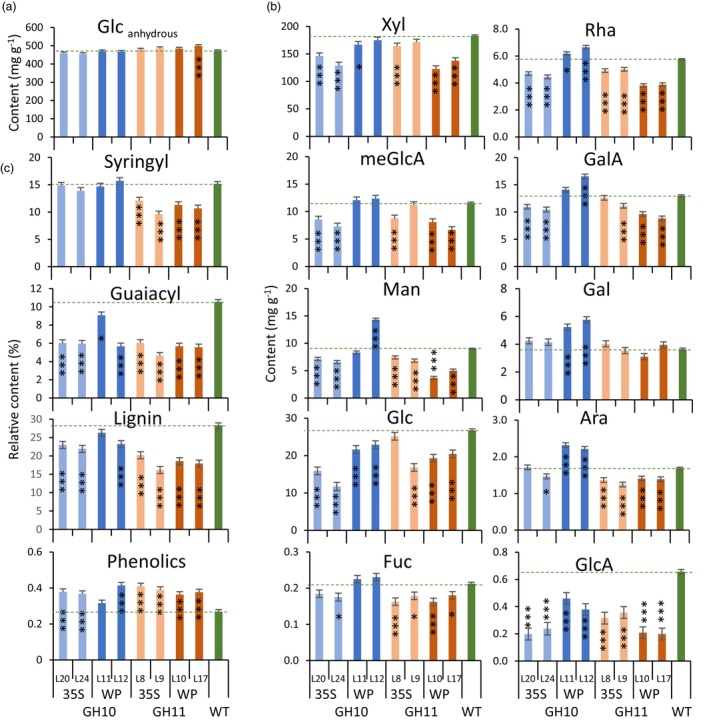
Chemical composition of wood in transgenic lines expressing GH10 and GH11 xylanases. (a) Glucan (anhydrous glucose) content in dry wood determined by sulfuric acid hydrolysis. (b) Matrix sugar content (hydrous) determined by methanolysis‐TMS per dry weight of dry destarched alcohol‐insoluble wood. (c) Relative content of syringyl (S) and guaiacyl (G) monolignols, total lignin (S + G + H) and phenolics in wood powder determined by the pyrolysis GC–MS. H – *p*‐hydroxyphenyl units. Data are means ± SE, *N* = 3 technical replicates of pooled material from 6 trees in a; *N* = 9 (3 technical and 3 biological replicates) in b; *N* = 3 biological replicates for c. *P ≤ 0.05; ***P ≤ 0.001 for comparisons with WT by Dunnett's test.

**Figure 4:**
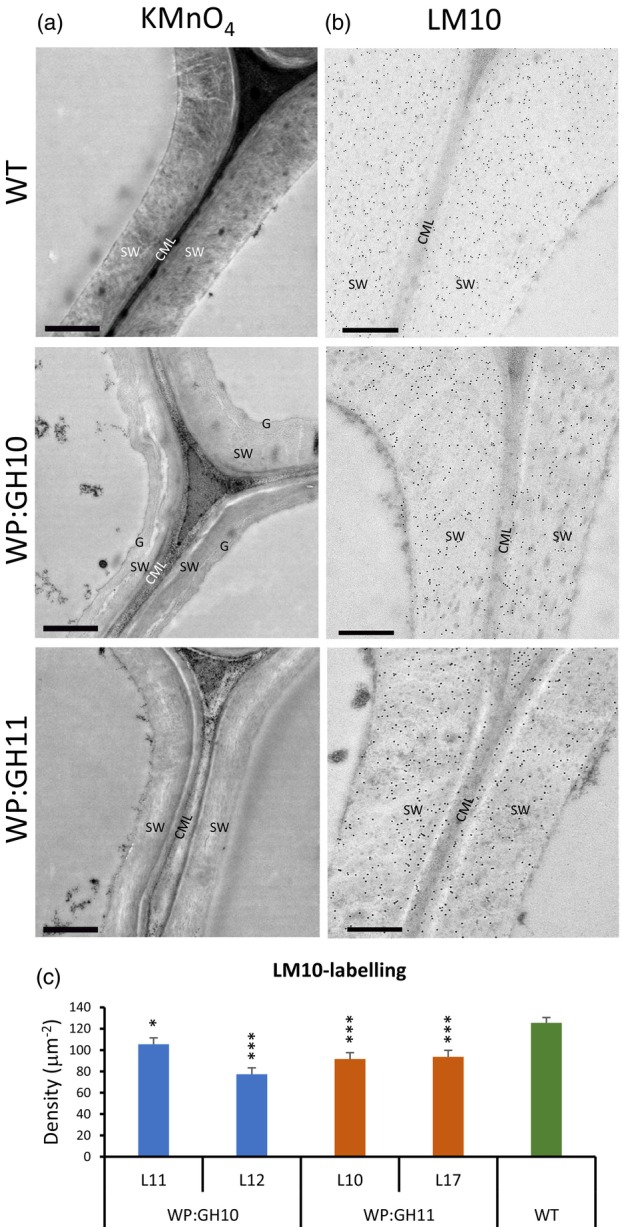
Transmission electron microscopy of cell walls in the xylem of transgenic lines expressing GH10 and GH11 xylanases showing differences in lignin and xylan content compared to wild type (WT). (a) Lignin in the fibre walls detected with KMnO_4_ which is seen as a dark deposit in the compound middle lamella (CML) and secondary walls (SW) is highly reduced in transgenic lines. Note also the presence of G‐layer (G) in one of the transgenic samples. (b, c) Immunogold localization of xylan in fibre cell walls using LM10 antibody (b) and quantification of gold particle density over secondary walls (c). Scale bar =1 μm in a and 500 nm in b; data in c are means ± SE, *N* = 2 trees × 3 images × 4 measurements. *P ≤ 0.05; ***P ≤ 0.001 for comparisons with WT by Dunnett's test.

The wood for the cell wall chemical analyses was collected from all xylanase‐expressing lines except for the WP:GH10_L13, because it was dwarf and did not produce much wood. Sulfuric acid hydrolysis showed no consistent changes in cellulose (glucan) content in transgenic lines (Figure 3a). On the other hand, acid methanolysis‐TMS analysis showed significant changes in matrix sugars (Figure 3b): xylose, MeGlcA and GlcA contents decreased in most or all transgenic lines, mannose contents decreased in most lines but WP:GH10, and most lines had lower glucose unit content than WT. WP:GH10 lines showed an increase in pectin‐related sugars including rhamnose, galacturonic acid, galactose and arabinose, whereas the opposite trend or no change was observed for other lines. Wood analysis by Py‐GC/MS revealed a significant decrease in total lignin and guaiacyl (G) unit contents in transgenic lines (Figure 3c). Although the G‐lignin units were substantially reduced in all transgenic lines, the syringil (S) lignin units were decreased only in GH11‐expressing lines. The content of other phenolics was on the other hand increased in the majority of the transgenic lines. The spatial distribution pattern of lignin and xylan in cell walls analysed by transmission electron microscopy revealed a severe depletion of lignin in the compound middle lamellae and SW layers of xylem fibres in transgenic trees (Figure 4a) and a significant decrease in gold particle density labelling LM10 xylan epitopes in transgenic lines (Figure 4b,c). Thus, cell wall analyses revealed major impact of xylanases on the lignin and xylan in wood cell walls.

### Detailed xylan analysis revealed that xylanases affected its molecular structure

**Figure 5:**
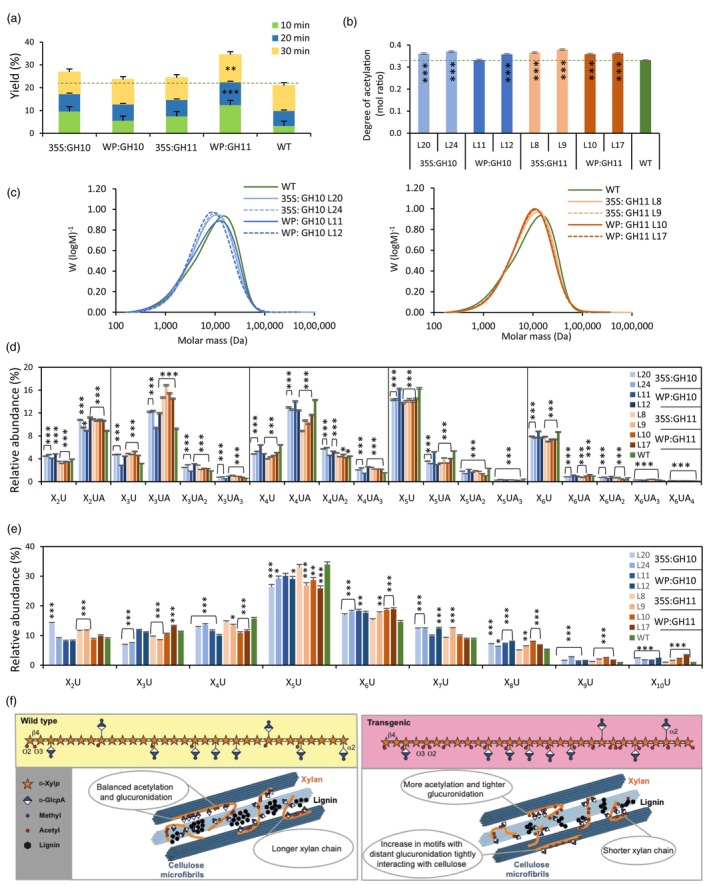
Characterization of xylan structure in transgenic lines expressing GH10 and GH11 xylanases. (a) Xylan yields during successive steps of subcritical water extraction relative to starting xylan weight. (b) The degree of acetylation of xylan extracted by SWE for 30 min. (c) Size exclusion chromatography of 30‐min SWE extract. (d, e) Oligomeric mass profiling (OLIMP) by ESI‐MS of SWE‐extracted glucuronoxylan (d) or alkali‐extracted glucuronoxylan (e) hydrolysed with GH30 glucuronoxylanase. (f) A model structure of glucuronoxylan motifs and their role in secondary cell wall architecture in WT and transgenic lines based on b–e and Sivan *et al*. (2024). Relative abundance of oligosaccharides in d and e are calculated from the total ESI‐MS intensities. Data in a, b, d, e are means ± SE, *N* = 2 lines in a, 2 technical replicates in b, and 3 technical replicates in d and e, *P ≤ 0.05; **P ≤ 0.01; ***P ≤ 0.001 for comparisons with WT by Dunnett's test.

The extractability of the matrix polysaccharides, mainly xylan, was evaluated by sequential subcritical water extraction (SWE). The yields of xylan in 20‐ and 30‐min subcritical water extracts (SWE) determined as sum of xylose and MeGlcA contents was increased in WP:GH11 lines indicating increased xylan solubility compared to WT (Figure 5a). The xylan from 30‐min extracts of transgenic lines was characterized by a higher degree of acetylation (Figure 5b). The molar mass distribution of the 30‐min SWE extracts determined by size exclusion chromatography revealed a decrease in molecular weight in transgenic trees indicating the reduction in the degree of polymerization of xylan according to the expected cleavage activity of expressed xylanases (Figure 5c).

The oligomeric mass profiling (OLIMP) of acetylated xylan from SWE and digested with GH30 glucuronoxylanase showed an increased population of oligomers representing closer glucuronidation spacing and higher acetyl substitution (X_2_UA, X_3_UA) and a decreased abundance of oligomers representing more spaced substitutions (X_4_UA, X_5_U, X_6_U) in transgenic lines (Figures 5d and S4). On the contrary, OLIMP of alkali‐extracted xylan showed in opposite a significant decrease in oligomers representing closer glucuronidation (X_3_U to X_5_U) and increase in those representing more spaced glucuronidation (X_6_U to X_10_U) (Figures 5e and S5). Altogether, as shown in our models, these data indicate that the xylan domains with close MeGlcA and acetyl substitution are protected from GH10 and GH11 xylanases expressed in transgenic lines and that some regions with highly spaced glucuronidation could be hindered from GH10 and GH11 xylanases either by a tight interaction with cellulose or by high acetyl substitution (Figure 5f).

### Xylanases‐induced cell wall chemical changes improved saccharification potential of wood

**Figure 6:**
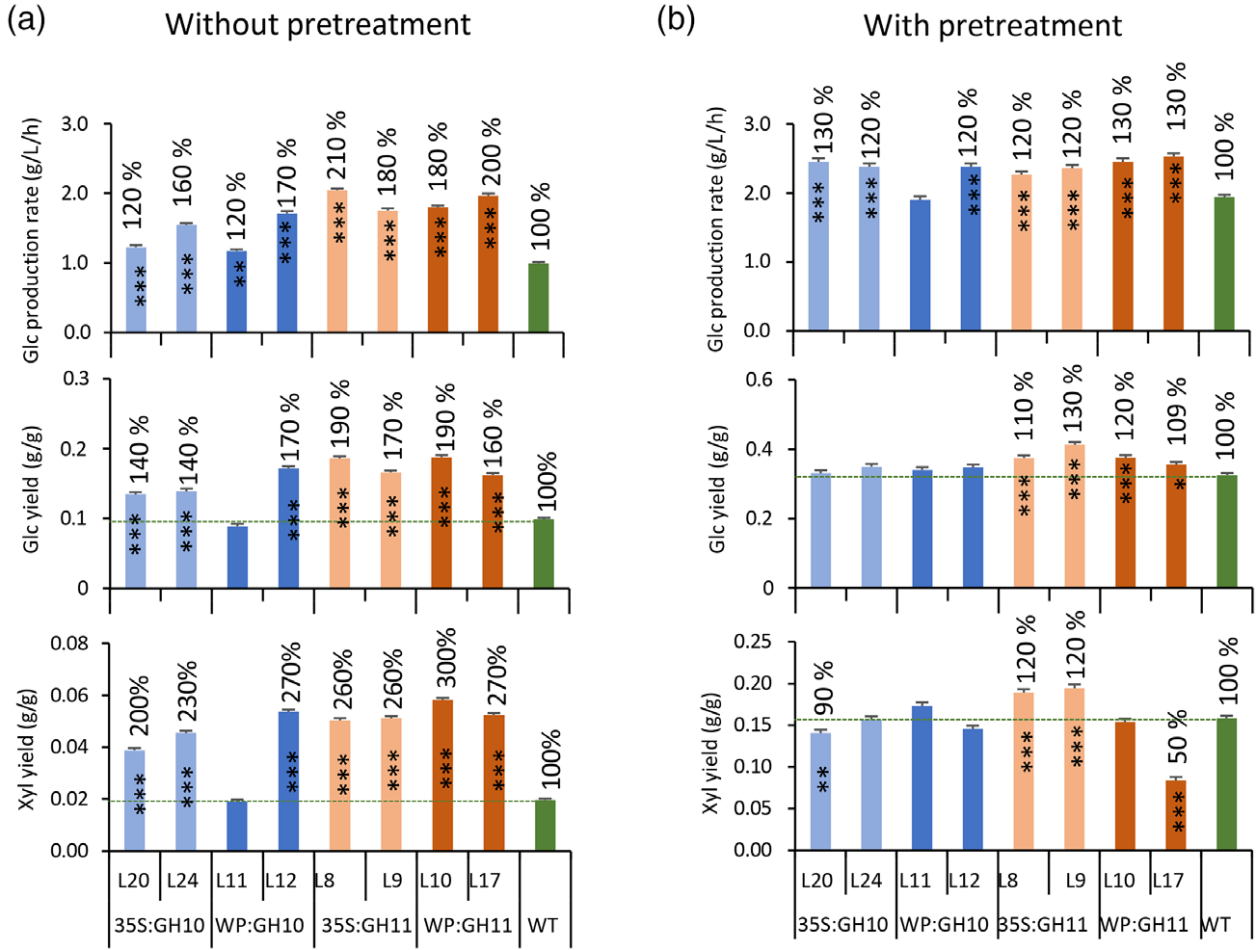
Effects of *in planta* expression of GH10 and GH11 xylanases on saccharification of wood. Glucose production rates, glucose yield and xylose yield in saccharification without (a) and with (b) acid pretreatment. Data are means ± SE, *N* = 3 or 6 technical replicates from the pooled material of 6 trees for transgenic lines and WT, respectively. *P ≤ 0.05; **P ≤ 0.01; ***P ≤ 0.001 for comparisons with WT by Dunnett's test.

Wood of xylanase‐expressing lines showed substantial reduction of recalcitrance which was particularly evident in saccharification without pretreatment. Glucose production rate, glucose yield and xylose yield increased up to 210%, 190% and 300% of WT levels, respectively (Figure 6a). The improvements for GH11 were more substantial compared to GH10 (even when disregarding line WP:GH10_11), as supported by *P*_contrast GH10 *vs* GH11_ ≤ 0.0001 for all three parameters. After pretreatment, glucose production rates were also increased in transgenic lines, but to a lesser extent (up to 130%), whereas only GH11 lines showed higher glucose yields (up to 130%) than WT (Figure 6b). Total xylose yields were increased for some lines (35S:GH11) but reduced for others (35S:GH10 and WP:GH11) reflecting the net decrease in xylose unit content of these lines. Furthermore, the yields of mannan and galactan in pretreatment liquid were altered in many transgenic samples reflecting changes in their content and solubility (Figure S6).

### Changes in hormonomics and metabolomics provide evidence for intrinsic regulation of vascular differentiation and cell wall lignification in transgenic trees

**Figure 7:**
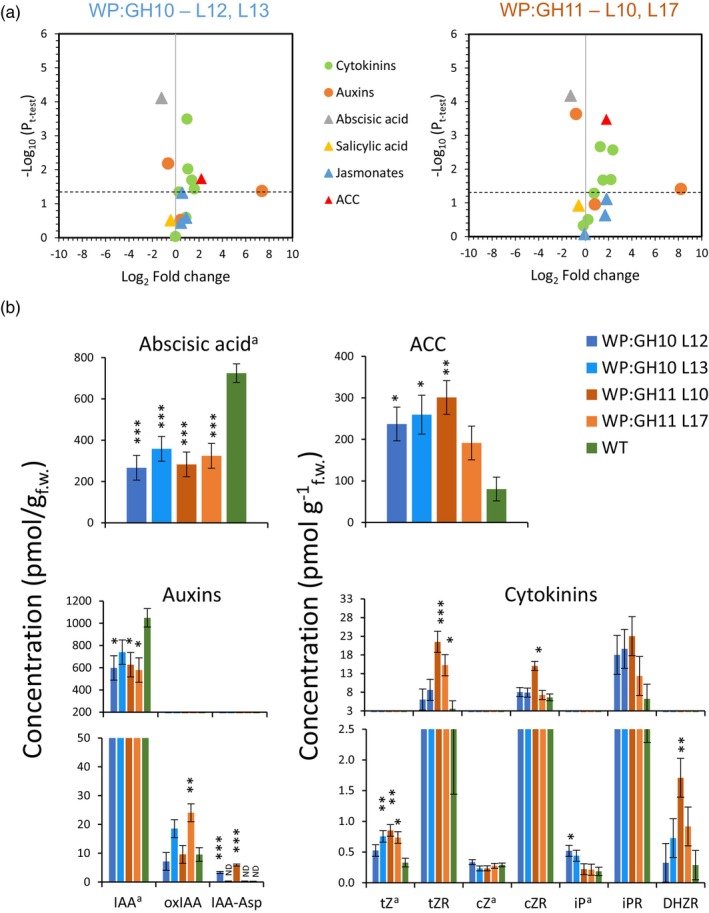
Changes in hormonal status in wood‐forming tissues of transgenic lines expressing GH10 or GH11 xylanases. (a) Volcano plots showing all detected hormones. Coloured signs above the dashed lines show the hormones that were either significantly increased or reduced in the transgenic lines at *P* ≤ 0.05 compared with wild type (WT). (b) Bar plots showing mean contents of abscisic acid, ACC, auxins and cytokinins in transgenic lines as compared to WT. Data are means ± SE, *N* = 4 trees for transgenic lines and 7 for WT; *P ≤ 0.05; **P ≤ 0.01; ***P ≤ 0.001 for comparisons with WT by Dunnett's test. ND, not detected; active hormones are marked with “a”. ACC, 1‐aminocyclopropane‐1‐carboxylic acid; IAA, indole‐3‐acetic acid; oxIAA, 2‐oxoindole‐3‐acetic acid; IAA‐Asp, IAA‐aspartate; *t*Z, *trans*‐zeatin; *t*ZR, *trans*‐zeatin riboside; *c*Z, *cis*‐zeatin; *c*ZR, *cis*‐zeatin riboside; iP, N^6^‐isopentenyladenine; iPR, N^6^‐isopentenyladenosine; DHZR, dihydrozeatin riboside.

To understand the mechanisms of developmental changes triggered by xylan integrity impairments in SWs, we analysed hormones in developing wood of WP:GH10 and WP:GH11 lines and WT. There was an overall similarity in hormonal changes induced by GH10 and GH11, with many cytokinin forms, some auxin forms, abscisic acid (ABA) and the ethylene precursor 1‐aminocyclopropane‐1‐carboxylic acid (ACC) being significantly affected, whereas no changes were seen in jasmonates (JA) or salicylic acid (Figure 7a). Significant increases in active forms of cytokinins, trans‐zeatin and N^6^‐isopentenyladenine, and their riboside precursors were evident indicating elevated cytokinin signalling (Figure 7b). In contrast, the levels of indole‐3‐acetic acid (IAA) were decreased with concomitant increases in inactivated IAA forms. The ABA showed a two‐fold decrease while ACC concentration increased almost four times in transgenic trees, indicating altered stress signalling *via* ABA and ethylene. This provides evidence that hormones regulating the cambial activity, xylem differentiation and stress responses were affected by the expression of xylanases in xylem cells forming SWs.

**Figure 8:**
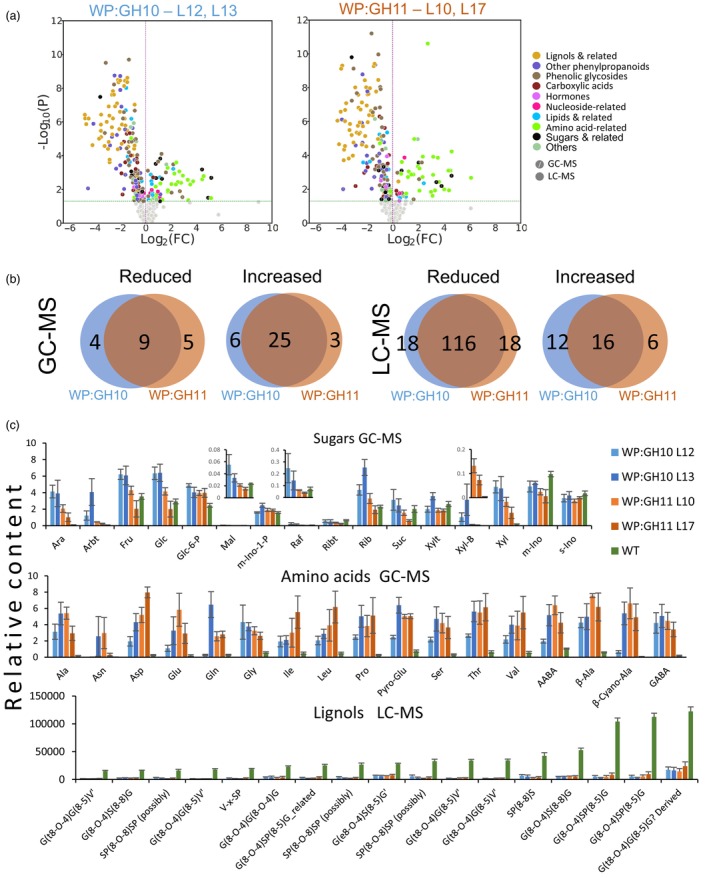
Metabolomes of developing wood in transgenic lines expressing xylanases show massive changes in several groups of compounds. (a) Volcano plots of metabolites analysed by LC–MS and GC–MS showing groups of compounds significantly affected (*P* ≤ 0.05, *t*‐test) in transgenic lines compared to wild type (WT). (b) Venn diagrams showing number of metabolites significantly affected in transgenic lines compared to WT. (c) Quantitative variation in integrated peaks (in relative units) corresponding to the most affected groups of compounds (amino acids, sugars and most abundant lignols). Data are means ± SE, *N* = 8 trees for WT and 4 for transgenic lines. Complete lists of metabolites are shown in Tables S1–S3.

Xylanases induced striking changes in the metabolomes of transgenic lines, which were highly similar between WP:GH10 and WP:GH11 lines as shown by the volcano plots and Venn diagrams (Figure 8a,b). Significantly affected compounds detected by GC–MS analysis were mostly upregulated. They comprised amino acids and sugars, including xylose (Xyl) and xylobiose (Xyl‐B) (Figure 8c). LC–MS analysis revealed metabolites mostly reduced in transgenic lines of which the most affected were lignols (some with over 30‐fold decrease), phenolic glycosides and other phenylpropanoid‐related metabolites (Figure 8a–c), demonstrating the specific impact on lignin biosynthetic pathway.

### Transcriptomic changes

#### Overview of transcriptomic changes in WP:GH10 and WP:GH11 transgenic lines

**Figure 9:**
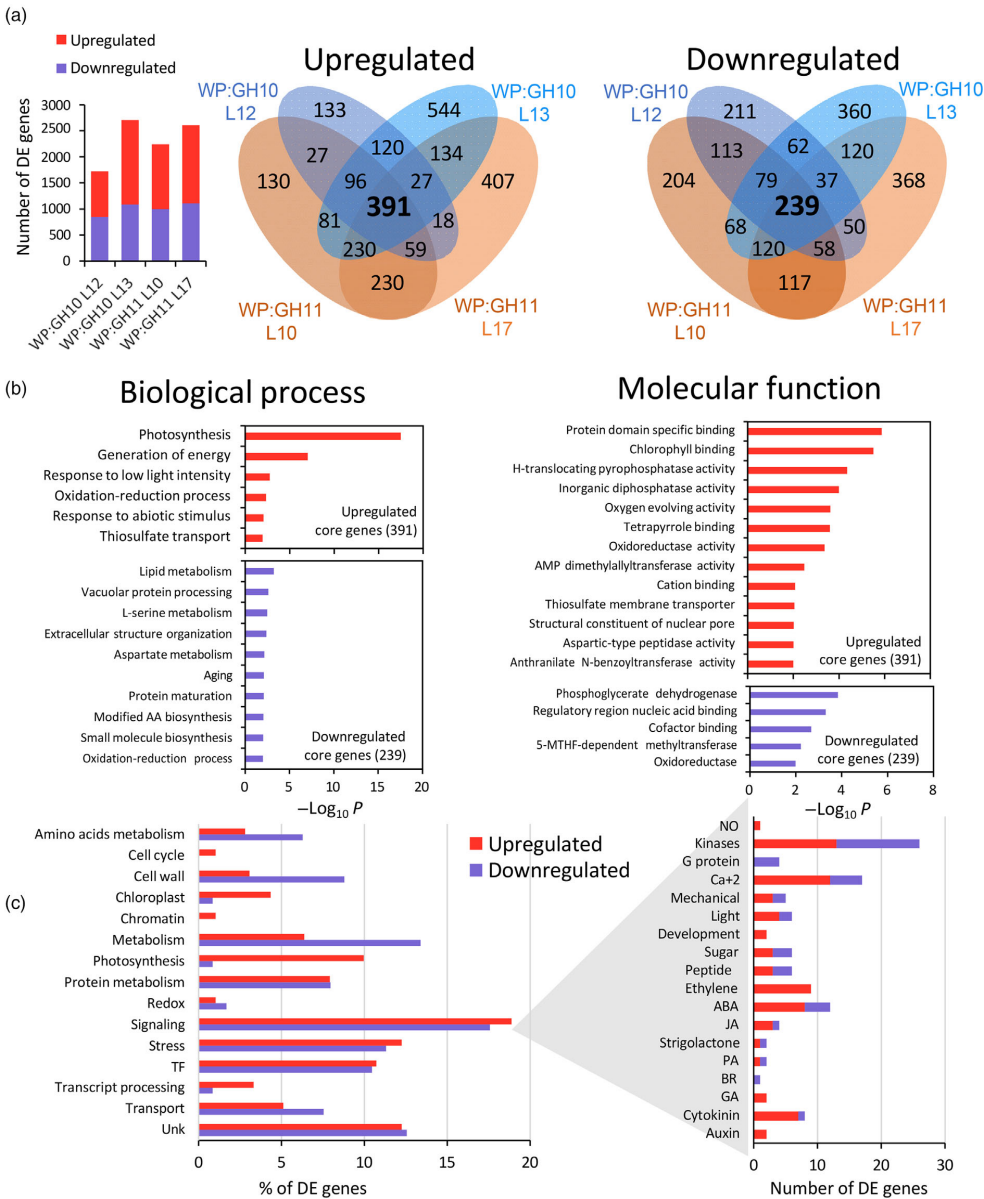
Expression of GH10 and GH11 xylanases alters transcriptomes of wood‐forming tissues in transgenic lines. (a) Numbers of differentially expressed (*P*_adj_ ≤0.01, |Log_2_(fold change)| ≤ 0.58) genes and associated Venn diagrams. Number of core genes differentially expressed in both transgenic lines of each construct are shown in bold. (b) Gene ontology (GO) enrichment analysis for the core DE genes. (c) DE genes by different functions to which the genes were exclusively assigned. The functional classification is listed in Table S7. 5‐MTHF, 5‐methyltetrahydrofolate; AA, amino acid; ABA, abscisic acid; BR, brassinosteroids; GA, gibberellins; JA, jasmonate; NO, nitric oxide; PA, polyamines; TF, transcription factor.

RNA‐seq analysis of developing xylem identified 1600–2700 differentially expressed genes (DEG) in WP:GH10 and WP:GH11 lines, with an overrepresentation of upregulated genes (Figure 9a, Table S4). The core genes affected in common for all xylanase‐expressing lines included 391 upregulated and 239 downregulated genes (Table S5). Gene ontology (GO) enrichment analysis of these genes revealed upregulation in GO terms related to the photosynthesis, chlorophyll binding, generation of energy and stress and downregulation in categories related to lipid, protein and amino acid metabolism, oxidoreductase activities, and cell wall biosynthesis (Figure 9b, Table S6).

The exclusive functional classification of the core genes (Figure 9c, Table S7) showed that signalling, stress and transcription factors functions were most highly represented among both up‐and downregulated genes. Interestingly, many of the stress‐related genes were annotated as responsive to anoxia. “Amino acid metabolism” and “cell wall” categories were highly represented among the downregulated genes whereas about 10% of upregulated genes were associated with photosynthesis.

#### Transcriptomic changes in signalling and stress response genes

Since the signalling was the most represented function among DEGs, we analysed these genes in more detail. Transcripts for different kinases and calcium signalling genes were the two most highly represented groups in the signalling category (Figure 9c, Table 1). Of the hormone‐related genes, those related to ABA, ethylene, and cytokinins were most highly represented, which is in good agreement with the hormone analyses.

**Table 1:** Signalling‐related genes significantly up‐ (U) or downregulated (D) in common by GH10 and GH11 xylanases

Category	*P. tremula* v. 2.2 *Potra2n*	*P. trichocarpa v. 3.1 Potri*	Ath Diamond BLAST	Ath name	U/D	Category	*P. tremula* v. 2.2 *Potra2n*	*P. trichocarpa v. 3.1 Potri*	Ath Diamond BLAST	Ath name	U/D
ABA	7c15809	007G101700	AT5G63910	*AtFCLY*	U	GA	5c10699	005G239100	AT1G75750	*AtGASA1*	U
ABA	13c26213	013G009800	AT3G58450	*AtGRUSP/USP*	U	JA	16c29395	016G017900	AT3G22160	*AtJAV1*	U
ABA	8c17204	008G065000	AT2G39980		U	JA	432s35660	001G015500	AT3G45140	*AtLOX2*	U
ABA	5c12343	005G058200	AT2G38820		U	JA	10c21496	010G108200	AT3G17860	*AtJAZ3*	U
ABA	6c15263	006G014500	AT5G47550	*AtCYS5*	U	Kinases	1069s36958	005G014700	AT2G19130		U
ABA	9c18899	009G139400	AT4G38470	*AtSTY46*	U	Kinases	743s36657	005G014700	AT2G19130		U
ABA	9c19650	009G054000	AT1G50920	*AtNOG1‐1*	U	Kinases	3c7707	003G090100	AT4G35470	*AtPIRL4*	U
ABA	1c767	001G092500	AT5G53160	*AtRCAR3*	U	Kinases	11c22893	011G110200	AT1G78850	*AtGAL2, MBL1*	U
ABA	18c32305	018G107800	AT5G46220	*AtTOD1*	D	Kinases	12c24522	012G097000	AT3G48530	*AtKING1*	U
ABA	4c9033	004G067400	AT1G73390		D	Kinases	18c32838	018G048100	AT5G21940		U
ABA	17c31097	017G094500	AT1G13740	*AtAFP2*	D	Kinases	6c13385	006G219800	AT5G21940		U
ABA	6c13197	018G043500	AT3G63520	*AtNCED1/CCD1*	D	Kinases	8c18059	008G156000	AT3G22750		U
Auxin	3c7907	003G063400	AT1G54200	*AtBG3*	U	Kinases	12c24080	012G043200	AT1G73500	*AtMAPKK9*	U
Auxin	1c3599	001G410400	AT5G54510	*AtDFL1/GH3.6*	U	Kinases	4c9196	004G086000	AT5G37660	*AtPDLP7*	U
BR	5c11696	005G124000	AT3G50660	*AtDWF4*	D	Kinases	158s34765	019G050500	AT5G16810		U
Ca^2+^	2c6269	002G019800	AT1G20080	*AtSYTB*	U	Kinases	15c28339	015G094700	AT3G48530	*AtKING1*	U
Ca^2+^	6c14829	006G062800	AT1G30270	*AtCIPK23*	U	Kinases	2c6154	002G032100	AT1G59580	*AtMPK2*	U
Ca^2+^	14c27171	014G104200	AT1G01140	*AtCIPK9*	U	Kinases	8c17227	008G067900	AT2G40090	*AtATH9*	D
Ca^2+^	19c33304	019G128100	AT2G30360	*AtCIPK11*	U	Kinases	8c17956	008G144900	AT1G10850		D
Ca^2+^	7c16155	007G055100	AT5G67480	*AtBT4*	U	Kinases	2c6116	002G036200	AT1G03920	*AtNDR5*	D
Ca^2+^	1c1997	001G231100	AT2G44310		U	Kinases	10c20345	010G231000	AT3G52790		D
Ca^2+^	18c33019	018G027300	AT4G32300	*AtSD2‐5*	U	Kinases	11c23439	011G034900	AT1G61400		D
Ca^2+^	3c6844	003G181900	AT5G25110	*AtCIPK25*	U	Kinases	3c7017	003G164200	AT1G03080	*AtNET1D*	D
Ca^2+^	114 s34562	015G126800	AT5G62390	*AtBAG7*	U	Kinases	6c13382	006G220100	AT2G26330	*AtER*	D
Ca^2+^	1c19	001G002000	AT1G55500	*AtECT4*	U	Kinases	10c20401	010G225300	AT2G39110	*AtPBL38*	D
Ca^2+^	6c15264	006G014400	AT3G50950	*AtZAR1*	U	Kinases	8c18004	008G150200	AT3G05990	*AtLLR3*	D
Ca^2+^	3c6476	003G222700	AT3G13460	*AtECT2*	U	Kinases	203s34959	007G039800	AT5G66850	*AtMAPKKK5*	D
Ca^2+^	17c31919	017G000900	AT4G02600	*AtMLO1*	D	Kinases	2c5709	002G077900	AT1G77280		D
Ca^2+^	4c10336	004G218500	AT1G11000	*AtMLO4*	D	Kinases	2c6370	002G009400	AT1G76360	*AtPBL31*	D
Ca^2+^	8c17563	008G103900	AT5G49480	*AtCP1*	D	Kinases	4c10446	004G231600	AT5G49760	*AtHPCA1*	D
Ca^2+^	6c13702	006G187500	AT4G30993		D	Light	5c11650	005G130700	AT5G66560		U
Ca^2+^	4c10186	004G202200	AT2G27480		D	Light	7c15989	005G090000	AT5G04190	*AtPKS4*	U
Cytokinins	6c13614	006G196900	AT1G15670	*AtKMD2*	U	Light	13c24949	013G159000	AT2G30520	*AtRPT2*	U
Cytokinins	10c21288	008G117100	AT1G13260	*AtRAV1*	U	Light	5c11293	005G175800	AT1G21920	*AtMORN3*	U
Cytokinins	10c22148	010G030500	AT5G19040	*AtIPT5*	U	Light	11c22895	011G109900	AT2G42610	*AtLSH7, LSH10*	D
Cytokinins	5c11275	005G177600	AT1G21830		U	Light	13c26086	013G024400	AT5G64330	*AtDOT3/NPH3*	D
Cytokinins	9c19633	009G055800	AT1G01550	*AtBPS1*	U	Mech.	243s35083	T092400	AT3G51660	*AtMDL3*	U
Cytokinins	8c18492	008G202200	AT5G19040	*AtIPT5*	U	Mech.	3c7622	003G099800	AT1G32090	*AtOSCA1.8*	U
Cytokinins	15c29002	015G023200	AT4G27950	*AtCRF4*	U	Mech.	19c33668	019G079300	AT1G72160	*AtPATL3*	U
Cytokinins	15c28518	015G078200	AT5G62960	*AtARR6*	D	Mech.	5c11148	002G069500	AT1G43700	*AtVIP1/SUE3*	D
Development	15c28320	015G096500	AT3G48550		U	Mech.	8c17949	008G144300	AT2G02170		D
Development	14c26462	014G019500	AT4G24220	*AAtVI31/VEP1*	U	NO	7c15899	007G092300	AT5G65030		U
Ethylene	19c34043	T069600	AT5G39890	*AtHU43/PCO2*	U	PA	1c3393	001G388900			U
Ethylene	4c9202	004G086600	AT2G38540	*AtLTP1/LP1*	U	PA	3c7590	003G103600	AT1G31830	*AtPUT2/PQR2*	D
Ethylene	11c22474	011G156200	AT1G06650		U	Peptides	18c32791	018G057100	AT5G25930	*AtHSL3/NUT*	U
Ethylene	17c30762	017G135800	AT1G06620		U	Peptides	10c20934	010G169300		*AtCLE45*	U
Ethylene	8c18146	008G164400	AT3G23150	*AtETR2*	U	Peptides	3c7873	003G074000	AT1G72300	*AtPSY1R*	U
Ethylene	18c32128	018G130800	AT5G25350	*AtEBF2, EBF1*	U	Peptides	3c6613	003G206000	AT5G12950	*AtPAF1*	D
Ethylene	1c65	001G007100	AT3G13610	*AtDLO2*	U	Peptides	154s34742	003G206000	AT5G12950	*AtPAF1*	D
Ethylene	16c29776	016G059700	AT3G58040	*AtSINAT2*	U	Peptides	3c6614	003G206000	AT5G12950	*AtPAF1*	D
Ethylene	1c3337	001G381700	AT1G17020	*AtSRG1*	U	Peptides	14c26580	014G034500			D
Ethylene	2c5831	002G065600	AT5G50080	*ERF110*	U	SL	2c5333	002G118900	AT3G03990	*AtD14*	U
Ethylene	2c4582	002G201600	AT3G16770	*ERF72, RAP2.3*	U	SL	8c17236	008G069100	AT2G40130	*AtSMXL8*	D
Ethylene	14c27376	014G126100	AT3G16770	*ERF72, RAP2.3*	U	Sugar	4c8814	004G047100	AT1G28330	*AtDYL1/DRM1*	U
Ethylene	1c1345	001G154100	AT4G17500	*ERF1*	U	Sugar	1c105	001G010700	AT3G45240	*AtGRIK1*	U
G proteins	2c4810	002G175700	AT1G01200	*RABA3*	D	Sugar	1c1918	001G220800	AT5G21170	*AtKINBETA1*	U
G proteins	18c32613	018G075300	AT5G19610	*AtGNL2*	D	Sugar	14c27762	014G167400	AT5G21170	*AtKINBETA1*	D
G proteins	14c27157	014G102200	AT1G01200	*AtRABA3*	D	Sugar	7c15922	007G089200	AT1G78020	*AtFLZ6*	D
G proteins	2c4342	002G231900	AT2G43120	*AtPRN2*	D	Sugar	2c5564	002G092900	AT1G78020	*AtFLZ6*	D
GA	1c3303	001G378400	AT1G78440	*AtGA2OX1*	U						

#### Transcriptomic changes in secondary wall‐related genes

To find out if SW formation was affected by the xylanases at the transcript levels, we identified differentially regulated genes in transgenic lines with 35S:GH10, WP:GH10 and WP:GH11 constructs among known SW‐related genes expressed in the wood‐forming tissues (Table S8). Among cellulose‐related genes, several genes from family GH9 encoding cellulases were found downregulated (Table 2). Among xylan‐related genes, those involved in the MeGlcA substitution (*PtGUX1‐A, PtGUX4‐A* and *PtGXM1*), and acetylation (*PtXOAT1*, *PtRWA‐A* and *PtRWA‐B*) were found downregulated. Lignin biosynthesis pathway was also affected due to downregulation of genes involved in monolignol biosynthesis (*PtPAL4, PtCAld5H2*, homologue of *AtCAD6, Pt4CL3* and *5, PtCCoAOMT1* and *2*) and polymerization (*PtLAC12/AtLAC17*). This indicates that specific programs modifying cellulose, responsible for xylan substitution and lignin biosynthesis were downregulated. Among the master switches regulating these programs in *Populus* (Ohtani *et al*., 2011; Zhong *et al*., 2010), we identified two *VND6* genes *PtVND6‐A2* and *PtVND6‐C2* genes (named after Li *et al*., 2012 as listed in Takata *et al*., 2019) and their downstream TFs, *PtMYB199* homologous to *AtMYB85 –* an activator of lignin biosynthesis, *PtSND2* and *PtNAC124* (homologous to *AtSND2*) and *PtMYB90*, *PtMYB161* homologous to *AtMYB52* activating cellulose and hemicellulose biosynthetic pathways (Schuetz *et al*., 2013; Zhong *et al*., 2008) downregulated in xylanase‐expressing lines (Table 2). This suggests that specific sub‐programs of SW biosynthesis have been downregulated via SW transcriptional cascade in transgenic lines. On the other hand, we also observed a strong downregulation of four *AtMYB4* homologues, including *PtLTF1* that regulates lignification in response to stress (Gui *et al*., 2019), upregulation of *PtMYB55* homologous to *AtMYB61* reported to positively regulate SW development in *Arabidopsis* coordinating a small network of downstream genes (Romano *et al*., 2012), and three homologues of *AtMYB73* involved in salinity stress response and lateral root development (Kim *et al*., 2013; Wang *et al*., 2021).

**Table 2:** Cell wall‐related genes significantly (*P*_adj_ ≤0.05) up‐ or downregulated in transgenic lines expressing GH10 and GH11 xylanases. Shown are values of Log_2_ fold change from WT levels. Values in bold are not significantly different from WT

*P. tremula* v. 2.2 Potra2n	*P. trichocarpa* v. 3.1	*Populus* name	Ath best BLAST	Ath name	Log_2_FC				Function
					WP:GH10		WP:GH11		
					L12	L13	L10	L17	
19c33790	Potri.019G069300	*PtGH9B3*	AT1G71380	*AtCEL3*	−0.65	−0.59	−0.81	−1.47	CEL
2c6226	Potri.002G023900	*PtGH9_28*	AT1G19940	*AtGH9B5*	−0.42	−1.29	−1.01	−0.99	
14c27669	Potri.014G157600	*PtGH9B11*	AT2G32990	*AtGH9B8*	−1.01	−1.33	−1.02	−1.21	
6c13189	Potri.006G240200	*PtGT43G*	AT1G27600	*AtIRX9‐L*	−0.55	−0.92	−0.72	−1.03	GX biosynthesis
7c15752	Potri.007G107200	*PtGUX1‐A*	AT3G18660	*AtGUX1*	−0.43	−0.84	−0.63	−0.67	
5c12551	Potri.005G033500	*PtGUX4‐A*	AT1G08990	*AtGUX5*	−0.88	−2.90	−2.33	−2.28	
3c8387	Potri.004G226800	*PtGXM1*	AT1G09610	*AtGXM1*	**−0.22**	−0.63	−0.34	−0.56	
14c26628	Potri.014G040300	*PtGATL1‐A*	AT1G19300	*AtPARVUS*	**−0.38**	−0.84	−0.72	−1.06	
1c2626	Potri.001G300800	*CE6*	AT4G34215	*AT4G34215*	−0.74	−0.87	−0.87	−0.89	GX acetylation
8c17245	Potri.008G069900	*PtXOAT1*	AT3G55990	*AtESK1*	−0.32	−0.60	−0.33	−0.60	
10c20767	Potri.010G187600	*PtXOAT2*	AT3G55990	*AtESK1*	−0.41	−0.67	**−0.25**	−0.52	
8c17246	Potri.008G070000	*PtXOAT6*	AT3G55990	*AtESK1*	**−0.48**	−1.45	−1.68	−1.37	
562s35967	Potri.001G376700	*PtXOAT8*	AT1G73140	*AtTBL31*	−0.38	−0.96	**−0.48**	−1.05	
1c3105	Potri.001G352300	*PtRWA_A*	AT2G34410	*AtRWA3*	−0.29	−0.83	−0.48	−0.70	
11c23099	Potri.011G079400	*PtRWA_B*	AT2G34410	*AtRWA3*	−0.42	−0.95	−0.58	−0.91	
10c20411	Potri.010G224100	*PtPAL4*	AT2G37040	*AtPAL1*	−0.50	−1.08	−0.57	−0.85	Lignin biosynthesis
16c29966	Potri.016G078300		AT4G37970	*AtCAD6*	−0.47	−0.51	−0.59	−0.62	
1c2649	Potri.001G304800	*PtCCoAOMT2*	AT4G34050	*AtCCoAOMT1*	−0.55	−1.15	−0.56	−0.60	
9c19246	Potri.009G099800	*PtCCoAOMT1*	AT4G34050	*AtCCoAOMT1*	−0.39	−0.77	−0.38	−0.56	
3c6783	Potri.003G188500	*Pt4CL5*	AT1G51680	*At4CL1*	−1.00	−1.80	−1.06	−0.67	
1c307	Potri.001G036900	*Pt4CL3*	AT1G51680	*At4CL1*	−0.50	−0.96	−0.52	−0.58	
6c14571	Potri.006G087500	*PtLAC12*	AT5G60020	*AtLAC17*	−0.10	−0.47	−0.45	−0.95	
1c3502	Potri.001G401300	*PtLAC7*	AT5G60020	*AtLAC17*	−0.54	**−0.48**	−0.35	−0.37	
7c15861	Potri.007G096200		AT2G22420	*AtPRX17*	1.59	2.42	1.04	**1.55**	PRX
6c14751	Potri.006G069600		AT2G41480	*AtPRX25*	1.83	2.37	1.69	1.50	
19c34464	Potri.T045500		AT4G33420	*AtPRX47*	−1.26	−2.19	−1.35	−0.88	
5c11750	Potri.005G118700		AT5G66390	*AtPRX72*	1.50	2.49	1.44	1.27	
16c30396	Potri.016G125000		AT5G64120	*AtPRX71*	4.63	3.76	**1.68**	5.00	
14c27236	Potri.014G111200	*PtMYB055*	AT1G09540	*AtMYB61*	**0.16**	0.72	0.82	0.72	Master switchers
2c5298	Potri.002G122600	*PtMYB177*	AT4G37260	*AtMYB73*	1.64	1.31	1.02	**0.56**	
5c11563	Potri.005G142600	*PtMYB029*	AT4G37260	*AtMYB73*	0.97	1.17	0.87	1.28	
611s36153	Potri.009G096000	*PtMYB019*	AT4G37260	*AtMYB73*	1.26	1.49	1.50	1.55	
19c34420	Potri.T011400		AT4G38620	*AtMYB4*	−1.28	−1.34	−1.38	−1.38	
4c9673	Potri.004G138000	*PtMYB093*	AT4G38620	*AtMYB4*	−0.96	−0.69	−0.74	−1.03	
4c9980	Potri.004G174400	*PtLTF1*	AT4G38620	*AtMYB4*	−1.59	−1.80	−1.38	−1.51	
9c18946	Potri.009G134000		AT4G38620	*AtMYB4*	−1.02	−1.04	−0.86	−0.94	
12c24760	Potri.012G127700	*PtMYB199*	AT4G22680	*AtMYB85*	−0.66	−0.88	−0.51	−0.52	
11c23287	Potri.011G058400	*PtNAC124*	AT4G28500	*AtSND2*	−0.57	−0.75	−0.89	−0.96	
4c8837	Potri.004G049300	*PtSND2*	AT4G28500	*AtSND2*	−0.70	−0.96	−0.73	−0.80	
12c24750	Potri.012G126500	*PtVND6‐A2*	AT1G12260	*AtVND4*	−0.52	−0.51	−0.33	−0.33	
5c11773	Potri.005G116800	*PtVND6‐C2*	AT2G18060	*AtVND1*	−0.96	−0.47	**−0.34**	−0.87	
15c28915	Potri.015G033600	*PtMYB090*	AT1G17950	*AtMYB52*	**−0.30**	−0.68	−0.47	−0.72	
7c15498	Potri.007G134500	*PtMYB161*	AT1G17950	*AtMYB52*	−0.45	−0.87	−0.73	−0.82	

CEL, cellulase; GX, glucuronoxylan; PRX, peroxidase.

#### Co‐expression networks formed by genes commonly affected in WP:GH10 and WP:GH11 lines

**Figure 10:**
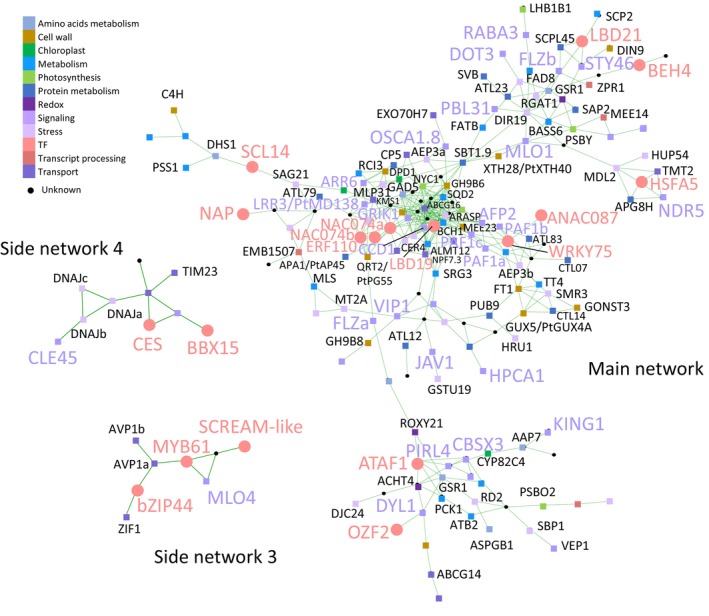
Co‐expression networks of the core genes differentially expressed in GH10‐ and GH11‐expressing aspen lines. Gene exclusive functional classification is indicated by the colours of the nodes, as listed in Table S7. Signalling‐ and stress‐related genes and transcription factors are shown on large coloured fonts. All gene names use *Arabidopsis* gene symbols, unless indicated by prefix Pt. For the gene expression data and all identified networks, please see Figures S7–S10.

To identify co‐expression networks of DEGs that might operate in developing wood, we used AspWood database (Sundell *et al*., 2017). Eight networks were identified, the main network and seven side networks, and expression of the genes of each network in wood‐forming tissues, in different tree organs and in different xylanase‐expressing lines was illustrated as heatmaps (Figures 10 and S7–S10, Table S7). The main network included mostly genes expressed late during xylogenesis, but it contained smaller subnetworks of genes expressed during SW formation and during primary wall stage of xylem differentiation (Figure S7). It was dominated by signalling‐ and stress‐related genes (Figure 10, Table S7), including many kinases, calcium signalling components, genes related to hormones (peptide – *PAF1s*, ethylene, ABA – *AtAFP2*, and *AtNCED1*, cytokinin – *AtARR6*), sugar responses, and stress (dehydration, salt, freezing and anoxia). The highly interconnected transcriptional factors included upregulated *AtWRKY75, AtERF110/PtERF57* and *AtLBD21/PtLBD047* and downregulated *AtLBD19*/*PtLBD043* and several *AtNAC074* homologues. The cell wall‐related genes included downregulated *PtGH9B11, PtGH9_18, PtGUX4A*, and upregulated *AtXTH28/PtXTH40*.

Among the side networks, network 3 included genes differentially expressed in the cambium (Figures 10 and S8), with three transcription factors, *AtMYB61, AtSCREAM‐like* and *AtbZIP44*, the calcium‐signalling‐related gene *AtMLO4* and transporters *AtZIF1* and *AtAVP1*. Side networks 4 and 6 were grouping genes expressed in the phloem that were almost all upregulated in transgenic lines (Figures 10 and S9). Network 4 included *AtCLE45* encoding a peptide hormone and transcription factors *AtCES* and *AtBBX15*, whereas network 6 included a kinase (*AtMPK2*), peroxidase *AtPRX72* and stress‐related genes (*AtGSTF11* and *AtKTI5*).

The side networks 1 and 2 (Figure S8) grouped genes highly upregulated by xylanases, expressed in the leaves, phloem and mature xylem zone and involved in photosynthesis (*AtLHCA3, AtLHCB5, AtPSAD, AtPSAE, AtPSBR, AtPSBO2*) and photorespiration (*AtGOX1*).

Side networks 5 and 7 (Figures S9 and S10) grouped genes expressed at primary‐SW transition in developing xylem and low‐expressed in extraxylary tissues, which were downregulated except one, *AtZPR1* encoding LITTLE ZIPPER1. Network 7 included *AtSTP10* encoding hexose‐H^+^ proton symporter and *AtGNL2* encoding GNOM‐like 2 an ARF guanidine exchange factor regulating vesicle trafficking.

#### Unique transcriptomic changes induced by the two families of xylanases

Venn diagrams (Figure 9a) showed that 120 genes were jointly upregulated in two lines of WP:GH10 construct but were not affected in any of the lines of WP:GH11 construct whereas 230 genes were upregulated only in the GH11‐expressing lines. Similar analysis of downregulated genes revealed 62 specifically downregulated in WP:GH10 and 117 in GH11 lines. Thus, GH11 altered expression of approx. two times more genes than GH10 and these genes were more frequently upregulated (Table S9). GO analysis of these xylanase family‐specific genes (Table 10) revealed that GH11 induced more intense activation of stress and in particular raffinose stress‐related genes than GH10.

## Discussion

### Fungal xylanases reduced xylan content and altered its structure in aspen secondary walls

The majority of transgenic lines expressing fungal GH10 and GH11 xylanases in cell walls had reduced matrix polysaccharide xylose content (Figure 2b), and reduced molecular weight of SWE xylan (Figure 5c). This was accompanied by reduced signals from LM10 antibodies in SWs (Figure 4b,c) and increased content of soluble xylose and xylobiose (Figure 8c). Jointly these data demonstrate that the xylanases were active on cell wall xylan in aspen, cleaving the backbone. However, not all domains of xylan were equally susceptible to cell wall‐targeted GH10 and GH11 xylanases. Analysis of degree of acetylation (Figure 5b) and OLIMP profiles after GH30 glucuronoxylanase digestion of SWE xylan (Figure 5d) indicated that highly acetylated and tightly glucuronidated regions of xylan were less prone to hydrolysis by these xylanases as these substitutions are known to restrict xylan backbone digestion by GH10 and GH11 xylanases (Biely *et al*., 2016; Kojima *et al*., 2022). On the other hand, the enrichment in the oligosaccharide motifs with more spaced and even glucuronidation patterns (X_6_U to X_10_U) in the alkaline extracted xylan (Figure 5e) suggests that these populations, potentially interacting tightly with the cellulose surfaces in two‐fold screws, are not affected by the SW‐targeted fungal xylanases. All in all, these results indicate that the fungal xylanases target flexible and accessible xylan domains within the secondary wall, leaving the domains that probably interact tightly with cellulose and lignin (Figure 5f).

### Fungal xylanases affected wood cell wall structure and composition

An *irregular xylem phenotype* (*irx*) with thin‐walled fibres and collapsed vessels is typical for mutants impaired in the biosynthesis of SWs or one of its main components like cellulose, xylan and lignin (Brown *et al*., 2005; Hao *et al*., 2014; Jones *et al*., 2001; Persson *et al*., 2007; Turner *et al*., 2001). Here we show that the *irx* phenotype was also induced by SW‐targeted fungal xylanases (Figures 2c and S2). This SW thinning was observed without any change in cellulose content but with prominent decrease in lignin content and composition (Figure 3a,c). While all lines had reduced content of G‐lignin, only GH11‐expressing lines had lower S‐lignin content. Similarly, *Arabidopsis thaliana* expressing GH10 and GH11 xylanases had decreased lignin, especially G‐lignin content in secondary xylem (Barbut *et al*., 2024). Moreover, mutations in xylan biosynthetic genes were also observed to lead to the same lignin defects (Barbut *et al*., 2024; Hao *et al*., 2014) suggesting an interdependence of lignification on xylan in SWs.

Whereas one mechanism of this interdependence has been proposed *via* xylan acting as a nucleation site for the polymerization of monolignols (Sapouna *et al*., 2023) our data point to a different possibility. First, we observed a severe decrease in mono‐ and oligolignols as well as phenolic glycosides (Figure 8) suggesting severely decreased lignin monomer biosynthesis in xylanase‐expressing lines. If the polymerization was affected *via* reduced lignin nucleation sites, one would expect increased content of unpolymerized lignols instead. Second, we observed downregulation of transcripts of lignification‐specific *MYB*s and several genes involved in monolignol biosynthesis and polymerization (Table 2). Similarly, key lignin biosynthetic genes were downregulated in *irx8/gaut12* mutant (Hao *et al*., 2014). Moreover, two negative regulators of phenylpropanoid biosynthesis encoding F‐box proteins *At*KFB and *At*KMD2 targeting phenylalanine ammonia‐lyase for ubiquitination (Zhang *et al*., 2013) were upregulated in xylanase‐expressing lines (Table S7). It is noteworthy that *AtKMD2* was upregulated in xylobiose‐treated *Arabidopsis* (Dewangan *et al*., 2023) and in *ixr9* mutant (Faria‐Blanc *et al*., 2018) that was also hypolignified (Barbut *et al*., 2024; Petersen *et al*., 2012), making it a strong candidate for downregulation of phenylpropanoid pathway in xylan‐compromised plants.

Xylanase‐expressing aspen lines showed also downregulation of some key genes required for biosynthesis of MeGlcA and acetyl substitutions of GX (Table 2). This could be a reaction counteracting the changes in GX substitution induced by xylanases in developing SW. This specific downregulation of the subprogram of GX biosynthesis likely involves specific regulatory genes (Chen *et al*., 2019; Ohtani *et al*., 2011; Taylor‐Teeples *et al*., 2015; Zhong *et al*., 2011), possibly including *VND6* homologues, the first layer master switches, and/or members of the third layer master switchers that were downregulated in transgenic lines (Table 2). Some of these master switchers, like *Pt*MYB161 (Wang *et al*., 2020a) or *Pt*LTF1 (Gui *et al*., 2019), could also participate in a feedback regulation of the SW biosynthesis in response to stress. An additional layer of suppression of SW program in transgenic plants could be mediated by decreased ABA levels (Figure 7b) since ABA signalling is needed to activate NST1 by phosphorylation (Liu *et al*., 2021). Suppression of SW program has been induced by overexpressing *Pt*MAN6 mannanase in poplar, which generated manno‐oligosaccharides that were responsible for this effect (Zhao *et al*., 2013). Possibly, the xylo‐oligosaccharides generated by xylanases could have a similar effect or the expression xylanases facilitated release of active manno‐oligosaccharides. These observations are in line with our previous hypothesis based on observations in aspen with suppressed *PtGT43BC* expression that SW impairment is sensed by plants resulting in general shutdown of SW biosynthetic program (Ratke *et al*., 2018).

### Expression of fungal xylanases altered growth and vascular differentiation pattern in aspen

Modification of xylan backbone by SW‐targeted GH10 and GH11 xylanases led to a substantial decrease in the height and biomass of trees (Figure 1). The previous experiments suppressing xylan backbone biosynthesis by knocking down *PtGT43A* and *B* (Lee *et al*., 2011), *PtGT43B* and *C* (Ratke *et al*., 2018), *PtGT47C* (Lee *et al*., 2009) or *PtGAUT12* (Biswal *et al*., 2015; Li *et al*., 2011) reported in contrast either no or positive effects on growth in *Populus*, especially when WP promoter was used. Intriguingly, the xylanases affected cambial growth by specifically inhibiting xylem formation and increasing phloem formation (Figure 1d), which was correlated with increased cytokinin content (Figure 7b). Phloem differentiation and cambial cell division are known to be regulated by local maxima in cytokinins which in turn exclude auxin maxima by regulating distribution of PIN transporters inhibiting auxin‐dependent activation of HD‐ZIPIII transcription factors and xylem differentiation (Bishopp *et al*., 2011; Haas *et al*., 2022; Immanen *et al*., 2016). Moreover, we found two homologues of *ZPR1* that is known to inactivate HD‐ZIPIII transcription factors (Wenkel *et al*., 2007), upregulated in xylanase‐expressing lines (Table S7), which would provide additional mechanism suppressing xylem formation. Therefore, modification of xylan in SW appears to positively affect mitotic divisions in the cambium, enhance phloem differentiation, and in case of xylanase‐expressing lines, inhibit xylem fate *via* transcriptional and hormonal regulation.

### Hormonal signalling pathways are affected in xylanase‐expressing lines

Impairment of xylan integrity in SW by fungal xylanases induced severe systemic changes such as reduced plant height (Figure 1a,b), reprogramming of cambial activity from xylem to phloem production (Figure 1d), and suppression of SW formation program in differentiating xylem (Table 2). Such changes require long‐ and short‐distance signalling, which likely starts in differentiating xylem cells and involves plant hormones.

Decrease in ABA content (Figure 7b) was supported by a downregulation of a key ABA biosynthetic gene, homologue of *AtNCED1*, and the decreased signalling by the upregulation of homologues of negative regulators of ABA signalling pathway: *AtATAF1* (Garapati *et al*., 2015) and *AtKING1* (Papdi *et al*., 2008) in xylanase‐expressing aspen (Tables 1 and S7). ABA forms a regulatory feedback loop with FERONIA (FER), a key RLK sensing cell wall integrity (Bacete and Hamann, 2020). ABA biosynthesis has been found to be downregulated after cell wall integrity signalling mediated by *At*THESEUS1 (*At*THE1) (Bacete *et al*., 2022), after stem mechanical disturbance (Urbancsok *et al*., 2023) and following *Botrytis cinerea* infection (Windram *et al*., 2012). On the other hand, ABA signalling was needed for increased biotic resistance in *Arabidopsis irx* mutants with defects in SW *CesA* genes (Hernández‐Blanco *et al*., 2007). In agreement with the well‐known antagonism between ABA and ethylene‐JA signalling pathways during different stresses, including the cell wall integrity impairment (Anderson *et al*., 2004; Bacete *et al*., 2020), there was evidence for activation of ethylene and JA signalling in xylanase‐expressing lines. The ACC levels were increased (Figure 7b) and there was strong upregulation of several ethylene‐related genes including *ETHYLENE RESPONSE FACTORS* (*ERF*s) which were also induced in xylobiose‐treated *Arabidopsis* (Dewangan *et al*., 2023). Among them was a homologue of *ERF1* regulating growth under stress (Hoang *et al*., 2020; Table 1 and S7). Upregulated JA biosynthesis and altered signalling were supported by the increased transcript levels of *AtLOX2*, *AtJAZ3* and *AtJAV1* homologues in transcriptome analysis. Both ethylene and JA signalling pathways were also stimulated in the developing xylem by mechanical stress (Urbancsok *et al*., 2023).

Strigolactones (SLs) and/or related carotenoids have been previously shown to mediate *irx* phenotype and freezing tolerance of *esk1/tbl29* and other SW mutants impaired in cellulose and xylan biosynthesis in *Arabidopsis* (Ramírez and Pauly, 2019). In xylanase‐expressing aspen lines the upregulation of a functional homologue of *AtDWARF14* (*AtD14*), *PtD14a*, encoding an SL receptor (Zheng *et al*., 2016) and *AtSMXL8 –* involved in feedback regulation of SL signalling (Wang *et al*., 2020b) – supports activation of signalling by SLs (Table 1). Moreover, a downregulation of a chalcone synthase transcript *AtTT4* (Table S7) which controls flavonoid biosynthesis downstream SLs (Richmond *et al*., 2022) was observed in common in xylanase‐expressing aspen and xylobiose‐treated *Arabidopsis* (Dewangan *et al*., 2023). Xylanases also induced *BYPASS1* (*BPS1*) (Table 1) encoding a plant‐specific inhibitor of a carotene‐related and xylem transported hormone inhibiting shoot development (Van Norman and Sieburth, 2007).

Upregulation of cytokinins in xylanase‐expressing aspen (Figure 7b) expectantly would increase plastid multiplication resulting in strong upregulation of photosynthesis‐related genes, and lipid and amino acid metabolism. Transcriptomics data supported these hypotheses with upregulation of a homologue of *AtPLASTID DIVISION2* (*AtPDV2)* that regulates plastid division (Chang *et al*., 2017) and many genes involved in plastid organization and photosynthesis (Figure 9b,c, Table S7). Among several cytokinin‐related DEGs, a homologue of *AtARR6* encoding a negative regulator of cytokinin response was downregulated (Table 1). *ARR6* has been implicated in cell wall modification and immunity (Bacete *et al*., 2020).

Thus, xylan integrity impairment caused by xylanases affected signalling *via* ABA, strigolactones/carotenes, ethylene and cytokinins, which overlaps with primary cell wall integrity signalling, and responses to mechanical and other abiotic and biotic stresses (Bacete and Hamann, 2020; Rivero *et al*., 2022).

### Local candidates for stress perception in secondary wall‐forming cells

The perception of xylan impairment in SW expectedly would involve local sensors including xylobiose (DAMP) sensors (Dewangan *et al*., 2023) and other cell wall integrity sensing components (Bacete and Hamann, 2020). One of them could be *HPCA1* (Tables 1 and S7) encoding a novel RLK responsible for H_2_O_2_ perception at the plasma membrane and activation of calcium influx (Wu *et al*., 2020). Several different calcium signalling‐related genes were upregulated, including mechanosensitive calcium channel *AtOSCA1.8* (Murthy *et al*., 2018; Yuan *et al*., 2014), defence‐activated calcium channel *AtZAR1* (Bi *et al*., 2021), *AtMDL3* known to be dependent on activity of mechanically activated MCA channels (Mori *et al*., 2018), *AtLBD38* regulated by calcium influx via cyclic nucleotide‐gated channel CNG15 (Tipper *et al*., 2023; Tables 1 and S7). Furthermore, *AtMLO4* and *AtMLO1* homologues were downregulated. *AtMLO4* is a calcium channel involved in mechanical stress and gravitropism signalling (Zhu *et al*., 2021). Among candidates expressed at primary‐SW transition other transporters were also identified, including *PtVP1.1* (*AtAVP1*) encoding a pyrophosphate‐fuelled proton pump regulating apoplastic pH and involved in stress responses (Yang *et al*., 2015), *AtSTP10* encoding a proton‐coupled sugar symporter responsible for uptake of monosaccharides from apoplast into plant cells (Bavnhøj *et al*., 2021), and *AtGNL2* involved in ER‐Golgi trafficking of proteins (Teh and Moore, 2007) (Figures 10, S8 and S10). Some of these genes were regulated in common with xylobiose‐treated *Arabidopsis* (Dewangan *et al*., 2023; Table S7).

### Xylanase‐induced changes in cell wall chemistry improved wood saccharification potential

Xylan binds to cellulose surfaces and interconnects lignin and was shown to impede enzymatic saccharification (DeMartini *et al*., 2013). Moreover, as it is the main source of yeast‐inhibiting acetic acid, it is predicted to inhibit fermentation (Donev *et al*., 2018). Therefore, decreasing xylan content and its modification are considered as effective strategies for improving biomass biorefinery properties. Here, we show that expressing either GH10 or GH11 xylanases in aspen SWs greatly improved glucose yield and production rate per wood weight in saccharification without pretreatment which were doubled or even tripled compared to WT (Figure 6). Previous experiments with *HvXyl1* expressed in poplar reported a 50% increase in glucose yield in saccharification after steam pretreatment (Kaida *et al*., 2009). Even milder xylan reduction by suppressing *GT43* genes of clades B and C resulted in increased in glucose yield in saccharification without pretreatment by 30% to 40%, but a negligible effect was observed after acid pre‐treatment (Lee *et al*., 2011; Ratke *et al*., 2018). The high glucose yields observed in the present study were however associated with growth penalties. On the other hand, no such penalties were observed in *GT43*‐suppressed aspen either in the greenhouse or in the field (Derba‐Maceluch *et al*., 2023; Ratke *et al*., 2015). It is therefore evident that saccharification benefits and growth are not necessarily negatively linked. Our current analysis of transcriptomic and metabolomic changes revealed many candidates for uncoupling regulation of growth and development from xylan reduction in xylanase‐expressing lines. Elucidation of their function could lead to designing better strategies to obtain saccharification‐improved plants that grow just as well as WT or even better.

## Conclusions

This study evaluated the effects of the post‐synthetic modification of xylan backbone by overexpression of fungal xylanases on growth, secondary cell wall characteristics and wood properties in aspen. Our results demonstrated that xylanases decreased the content of xylan and its molecular weight, and modified its substitution pattern. These structural changes inhibited tree growth, wood production, SW development and lignin biosynthesis. Hormonomics, metabolomics and transcriptomics analyses revealed that xylan impairment activated hormonal signalling and affected genetic regulatory pathways that modified cambial growth and adjusted the SW biosynthesis program, suggesting the activation of SW integrity sensing. Although the benefits of highly enhanced glucose yields in saccharification from transgenic wood biomass were offset by growth penalty, the identified candidates for the SW integrity sensing mechanism could be used to uncouple beneficial and undesirable effects for developing improved lignocellulose in aspen for biorefinery.

## Experimental procedures

### Generation of transgenic lines

The *Aspergillus nidulans* cDNA clones encoding GH10 (AN1818.2; GenBank: ABF50851.1) and GH11 (ANIA_03613; NCBI_GeneID:2873037, XP_661217.1) xylanases (Bauer *et al*., 2006) were used to generate expression vectors. The signal peptide of GH10 was replaced by the hybrid aspen (*Populus tremula* L. × *tremuloides* Michx.) signal peptide from gene *PtxtCel9B3* (alias *PttCel9B*) (GenBank AY660968.1; Rudsander *et al*., 2003) as described previously (Gandla *et al*., 2015), whereas native fungal signal peptide was used for GH11 vector. The cloning primers are listed in Table S11. The entry clones generated using the pENTR/D‐TOPO cloning system (Thermo Fisher Scientific, Uppsala, Sweden) were used to make the expression clones in either pK2WG7.0 (Karimi *et al*., 2002) for ectopic expression using 35S promoter or in pK‐pGT43B‐GW7 (Ratke *et al*., 2015) for expression specifically in cells developing secondary cell walls driven by the wood‐specific promoter (WP). The resulting vectors (35S:GH10, 35S:GH11, WP:GH10 and WP:GH11) were introduced into competent *Agrobacterium tumefaciens* Conn 1942, strain GV3101 using electroporation. Binary vectors were transformed into hybrid aspen (*Populus tremula* L. × *tremuloides* Michx., clone T89) as described previously (Derba‐Maceluch *et al*., 2015). Lines with the highest transgene expression were selected from 20 independent lines for further analyses.

### Plant growth in the greenhouse

*In vitro* propagated saplings were planted in soil (K‐jord, Hasselfors Garden AB, Örebro, Sweden) in 7 L plastic pots, watered to 25%–30% (v:v) soil moisture content, covered with transparent 8 L plastic bags, and grown for nine weeks in the phenotyping platform (WIWAM Conveyor, custom designed by SMO, Eeklo, Belgium) as described by Wang *et al*. (2022) under 18 h /6 h (day/night) light regime with 160–230 μmol/m^2^/s light intensity during the day, 22 °C/18 °C temperature, and the average air relative humidity of 60%. White light (FL300 LED Sunlight v1.1) and far‐red light (FL100 LED custom‐made, 725–735 nm) lamps from Senmatic A/S (Søndersø, Denmark) were used for illumination. After two weeks the bags were removed, and plants were watered automatically based on weight, their height was automatically measured.

At the end of experiment, trees were photographed, and stems' diameters at base and aboveground fresh weights were recorded. A 30 cm‐long stem segment above internode 37 was debarked, frozen in liquid nitrogen and stored at −70 °C for RNA, metabolomics and hormonomics analyses. The stem below was used for determining internode length. The 38th and 39th internodes were used for microscopy analyses. The four‐cm long bottom segment was used for SilviScan analysis, and the remaining stem was debarked and freeze‐dried for 48 h for wood chemistry analyses. Belowground biomass was determined by weighing cleaned and air‐dried roots.

### Wood microscopy analysis

For light microscopy, samples of three trees per line were fixed in FAA (4% formaldehyde, 5% acetic acid, 50% ethanol). Transverse sections (40–50 μm‐thick) were prepared with a vibratome (Leica VT1000S, Leica Biosystems, Nussloch, Germany) and stained with safranin‐alcian blue (Urbancsok *et al*., 2023). Lignin autofluorescence was analysed at 470 nm (Kitin *et al*., 2020). Images were acquired by Leica DMi8 inverted microscope (Leica Biosystems, Germany) equipped with digital camera and analysed with ImageJ software.

Another set of samples from the same trees were fixed in 0.1% glutaraldehyde, 4% paraformaldehyde, 50 mM sodium cacodylate buffer for 4 h at room temperature and embedded in LR white resin as described elsewhere (Pramod *et al*., 2014). Two μm‐thick sections were cut using an ultramicrotome (RMC Powertome XL, USA) and stained with toluidine blue O for light microscopy analysis. Transverse ultrathin sections (70–90 nm‐thick) were prepared using an ultramicrotome Ultracut E (Leica Biosystems) with a diamond knife and mounted on copper grids. For lignin localization, sections were stained with KMnO_4_ (Donaldson, 1992). The xylan immunogold labelling with LM10 monoclonal antibody was carried out as described by Pramod *et al*. (2014). All sections were examined with a transmission electron microscope (FEI TALOS L120C) at an accelerating voltage of 100 kV. Cell wall thickness and gold particle density were determined using ImageJ based on ten and twenty measurements per tree, respectively, for two lines per construct.

### SilviScan analyses

A SilviScan instrument (RISE, Stockholm, Sweden) was used for determining wood and fibre properties of six trees per line, 24 per WT as described by Urbancsok *et al*. (2023).

### Cell wall chemical analyses

For initial pyrolysis and TMS analyses, wood powder from three trees per line was obtained by filing the freeze‐dried wood and sieving the sawdust with Retsch AS 200 analytical sieve shaker (Retsch GmbH, Haan, Germany) to 50–100 μm.

Py‐GC/MS assay used 50 μg (±10 μg) of powder in a pyrolyser equipped with autosampler (PY‐2020iD and AS‐1020E, Frontier Lab, Japan) connected to a GC/MS (7890A/5975C, Agilent Technologies Inc., Santa Clara, CA, USA). The pyrolysate was processed and analysed according to Gerber *et al*. (2012).

Alcohol‐insoluble residue (AIR) was prepared as described by Gandla *et al*. (2015). AIR was destarched by α‐amylase (from pig pancreas, cat. nr. 10102814001, Roche, USA) and amyloglucosidase (from *A. niger* cat. nr.10102857001, Roche) enzymes and the matrix sugar composition was analysed by methanolysis‐trimethylsilyl (TMS) procedure as described by Pramod *et al*. (2021). The silylated monosaccharides were separated by GC/MS (7890A/5975C; Agilent Technologies Inc., Santa Clara, CA, USA) according to Gandla *et al*. (2015). Raw data MS files from GC/MS analysis were converted to CDF format in Agilent Chemstation Data Analysis (v.E.02.00.493) and exported to R software (v.3.0.2). 4‐*O*‐Methylglucuronic acid was identified according to Chong *et al*. (2013). The amount of monosaccharide units per destarched AIR weight was calculated assuming their polymeric form.

For the remaining cell wall analyses, the pith was removed from debarked and freeze‐dried stem segments the segments of seven trees per line were ground together using Retsch Ultra Centrifugal Mill ZM 200 (Retsch GmbH, Haan, Germany) equipped with a 0.5 mm ring sieve. The resulting wood powder was then sieved by Retsch AS 200 vibratory sieve shaker to isolate powder with particle size of 50–100 and 100–500 μm.

The 50–100 μm fraction was used in triplicates for monosaccharide analysis by a two‐step sulfuric acid hydrolysis (Saeman *et al*., 1954). In brief, 1 mg of sample was incubated with 125 μL of 72% H_2_SO_4_ at room temperature for 3 h, then diluted with 1375 μL of deionized water and incubated at 100 °C for 3 h. Hydrolysates were diluted 10 times with MilliQ water, filtered through 0.2 mm syringe filter (Chromacol 17‐SF‐02‐N) into HPAEC‐PAD vials and analysed by high‐performance anion exchange chromatography with pulsed amperometric detection (HPAEC‐PAD) (ICS‐6000DC, Dionex) equipped with a CarboPac PA1 column (4 × 250 mm, Dionex) at 30 °C using the eluent gradients previously reported (McKee *et al*., 2016). Quantification of monosaccharides was performed by standard calibration of ten monosaccharides (Ara, Rha, Fuc, Xyl, Man, Gal, Glc, GalA, MeGlcA and GlcA) with concentrations between 0.005 and 0.1 g/L.

For subcritical water extraction, 2 g of 100–500 μm wood powder was extracted with 0.2 M formate buffer, pH 5.0, at 170 °C and 100 bar in an accelerated solvent extractor (ASE‐300, Dionex, USA). Extraction proceeded in 4 steps with residence times of 10, 20, 30 and 60 min according to Sivan *et al*. (2023). Low‐molecular‐weight compounds were removed by dialysis using Spectra/Por 3 membranes (Spectrum, USA), and the extracted polymers were freeze‐dried.

For alkaline extraction, 1 g of wood powder with particle size 100–500 μm was incubated with 24% KOH for 24 h at room temperature (Escalante *et al*., 2012; Timell, 1961), filtered through 60 μm wire mesh and neutralized with 0.4 vol of acetic acid. Hemicellulose was precipitated with 96% ethanol (4 °C for overnight), centrifuged, washed in 80% ethanol, dissolved in distilled water and freeze‐dried. Molar mass of extracts was determined by size exclusion chromatography coupled to refractive index and UV‐detectors (SECurity 1260, Polymer Standard Services, Mainz, Germany). The samples (2 mg) were dissolved in 1 mL of dimethyl sulfoxide (DMSO Anhydrous, Sigma‐Aldrich) with 0.5% w/w LiBr (Anhydrous free‐flowing Redi‐Dri, Sigma‐Aldrich) at 60 °C, and filtered through 0.45 μm PTFE syringe filters (VWR). The separation was carried through GRAM Analytical columns of 100 and 10 000 Å (Polymer Standard Services, Mainz, Germany) at a flow rate of 0.5 mL/min and 60 °C. The columns were calibrated using pullulan standards between 345 and 708 000 Da (Polymer Standard Services, Mainz, Germany).

The acetyl content of water extracts was determined in duplicates by overnight saponification of approx. 5 mg of sample in 1.2 mL of 0.8 M NaOH at 60 °C with constant mixing, neutralization with 90 μL of 37% HCl and filtration through 0.45 mm Chromacol syringe filters (17‐SF‐02(N), Thermo Fisher Scientific). The released acetic acid was detected by UV at 210 nm using high‐pressure liquid chromatography with UV detector (Dionex‐Thermofisher Ultimate 3100, USA) and separation by a Rezex ROA‐organic acid column (300 × 7.8 mm, Phenomenex, USA) at 50 °C in 2.5 mM H_2_SO_4_ at 0.5 mL/min. Propionic acid was used as an internal standard.

For oligosaccharide mass profiling (OLIMP), the alkaline and 30 min water extracts were digested using GH10 endo‐β‐(1–4)‐xylanase from *Cellvibrio mixtus* (Megazyme), a GH11 endo‐1,4‐β‐xylanase from *Neocallimastix patriciarum* (Megazyme) and GH30 endo‐1,4‐β glucuronoxylanase (kindly provided by Prof. James F. Preston, University of Florida), incubating 1 mg of extract in 1 mL of 20 mm sodium acetate buffer (pH 5.5) and 10 U enzyme for 16 h at 37 °C. After enzyme inactivation at 95 °C for 10 min, the hydrolysates were ten times diluted in acetonitrile 50% (v/v) with 0.1% (v/v) formic acid and filtered through Chromacol 0.2 μm filters (Scantec Nordic, Sweden). Samples were then briefly passed through a ZORBAX Eclipse Plus C18 column 1.8 μm (2.1 × 50 mm) (Agilent Technologies, Santa Clara, CA) and the oligosaccharide profiles were analysed by HPAEC‐PAD as reported previously (McKee *et al*., 2016) using xylooligosaccharides (X_2_–X_6_; Megazyme) as external standards and electrospray ionization mass spectrometry (ESI‐MS) with a Synapt HDMS mass spectrometer (Waters, USA) in positive‐ion mode and capillary and cone voltage set to 3 and 70 kV, respectively. The oligosaccharides were detected as [M + Na] + adducts.

Oligosaccharide sequencing was achieved after the separation of labelled oligosaccharides by tandem LC‐ESI‐MS/MS. Derivatization was performed by reductive amination with anthranilic acid as previously described (Mischnick, 2012). The labelled oligosaccharides were separated through an ACQUITY UPLC HSS T3 column (150 × 2.1 mm, Waters, USA) at a flow rate of 0.3 mL/min and a gradient of increasing acetonitrile content (10%–30%) over 40 min. Mass spectrometric analysis was performed in positive mode with the capillary voltage and cone set to 3 and 70 kV, respectively. MS2 was performed by selecting the ion of interest [M + Na] + through single ion monitoring and subjecting it to collision‐induced dissociation using argon as the collision gas, at a ramped voltage of 35–85 V. Assignment of proposed structures was performed by reference to labelled standards and analysis of the fragmentation spectra using ChemDraw (PerkinElmer, Waltham, Massachusetts, USA).

### Saccharification assay

Three technical replicates from each line and six from WT were used for analytical‐scale saccharification. Wood powder moisture content was measured using Mettler Toledo HG63 moisture analyser (Columbus, OH, USA) and 50 mg of dry material was used per sample. Acid pretreatment was carried out using an Initiator single‐mode microwave instrument (Biotage Sweden AB, Uppsala, Sweden) with 1% (w/w) sulfuric acid at 165 °C for 10 min. Enzymatic hydrolysis without or after acid pretreatment was performed at 45 °C using 4 mg of the liquid enzyme mixture Cellic CTec2 (cat. nr. SAE0020, Sigma‐Aldrich, Saint Louis, MO, USA) as previously described (Gandla *et al*., 2021). Samples were analysed for glucose production rate at 2 h by using an Accu‐Chek® Aviva glucometer (Roche Diagnostics Scandinavia AB, Solna, Sweden) following the calibration with a set of glucose standard solutions. After 72 h, the yields of monosaccharides were quantified using HPAEC‐PAD (Ion Chromatography System ICS‐5000 by Dionex, Sunnyvale, CA, USA) (Wang *et al*., 2018).

### RNA analyses

Developing xylem tissues were scrapped from the debarked frozen stem and ground in a mortar with a pestle in liquid nitrogen. Approximately 100 mg of fine tissue powder was extracted with CTAB/chloroform:isoamylalcohol (24:1) followed by LiCl and sodium acetate/ethanol precipitation to isolate total RNA (Chang *et al*., 1993).

RNA samples from three trees per line were DNase treated with DNA‐free™ kit (cat. nr. AM1906, Thermo Fisher Scientific, Waltham, MA, USA) and then reverse‐transcribed using iScript™ cDNA synthesis kit (cat. nr. 1708891) (Bio‐Rad Laboratories, Hercules, CA, USA) following the manufacturers' instructions. Quantitative polymerase chain reactions (qPCRs) were performed using LIGHTCYCLER 480 SYBR GREEN I Master Mix (Roche, Indianapolis, IN, USA) in a Bio‐Rad CFX384 Touch Real‐Time PCR Detection System with 10 μL reaction volume. PCR program was 95 °C for 3 min, then 50 cycles of 95 °C for 10 s, 55 °C for 10 s and 72 °C for 15 s. UBQ‐L (Potri.005G198700) and ACT11 (Potri.006G192700) were selected as reference genes from four tested genes based on GeNorm (Vandesompele *et al*., 2002). The primer sequences are listed in Table S11. The relative expression level was calculated according to Pfaffl (2001).

For transcriptomics, RNA was purified as described previously (Urbancsok *et al*., 2023) and four or five biological replicates per transgenic line and eight biological replicates of the WT with RNA integrity number (RIN) ≥ 8 were used for cDNA preparation and sequencing using NovaSeq 6000 PE150 at Novogene Co., Ltd. (Cambridge, United Kingdom). Quality control and mapping to the *P. tremula* transcriptome (v.2.2), retrieved from the PlantGenIE resource (https://plantgenie.org; Sundell *et al*., 2015) were carried out by Novogene. Raw counts were used for differential expression analysis in R (v3.4.0) with the Bioconductor (v.3.4) DESeq2 package (v.1.16.1), as previously detailed (Kumar *et al*., 2019). The best BLAST hits were identified in *Populus trichocarpa* (v3.1) and *Arabidopsis thaliana* (v11.0).

### Hormonomics and metabolomics

Frozen developing xylem samples were ground as described above. Hormone profiling was done according to Simura *et al*. (2018), with slight modifications (Urbancsok *et al*., 2023). ACC (1‐aminocyclopropane‐1‐carboxylic acid) was quantified according to Karady *et al*. (2024).

Metabolites were extracted and analysed as described by Abreu *et al*. (2020) and Urbancsok *et al*. (2023) and processed by an untargeted approach. The generated data were normalized against the internal standard and weight of each sample. Changes in abundance between transgenic and WT samples were considered as significant if *P* ≤ 0.05 (*t*‐test) and |fold change| ≥ 1.5. The false discovery rate was <0.05.

### Statistical analyses

Unless otherwise stated, statistical analyses were performed in JMP Pro (v.16.0) software (SAS Institute Inc., Cary, NC, USA).

## Author contributions

PS performed majority of wood chemistry and microscopy analyses, interpreted the data and wrote the manuscript. JU processed wood material, extracted RNA, analysed transgene expression and prepared samples for omics analyses. JU, END and FRB carried out the greenhouse experiment and tree phenotyping. END performed bioinformatic analyses. MDM created transgenic aspen and collected samples for SilviScan analysis. ZY and GS carried out wood SilviScan analyses. JŠ, KC and MK analysed hormones. MLG and LJJ analysed saccharification potential. MM analysed metabolomics data. EH and FV carried out hemicellulose analyses. ERM designed cloning strategy. EJM designed and coordinated the research, secured the funding and finalized the paper with contributions from all authors.

## Supporting information


**Figure S1** Transgene expression levels in developing wood of transgenic lines expressing GH10 and GH11 xylanases based on RNA sequencing.
**Figure S2** Toluidine blue‐stained wood sections showing reduction in cell wall thickness and change in staining indicative of reduced lignin content in transgenic lines expressing xylanases.
**Figure S3** Fluorescence microscopy for detection of lignin in the wood tissue of transgenic lines expressing GH10 and GH11 xylanases.
**Figure S4** Oligomeric mass profiling (ESI‐MS) of acetylated glucuronoxylan extracted with 30 min subcritical water extraction from transgenic lines expressing GH10 and GH11 xylanases.
**Figure S5** Oligomeric mass profiling (ESI‐MS) of glucuronoxylan extracted with alkali from transgenic lines expressing GH10 and GH11 xylanases.
**Figure S6** Saccharification yields of mannose and galactose obtained from wood of transgenic lines expressing GH10 and GH11 xylanases.
**Figure S7** Main co‐expression network of the core genes differentially expressed in both lines expressing GH10 and both lines expressing GH11 xylanases in the wood‐forming tissues and their expression patterns in different tissues and transgenic lines.
**Figure S8** Side co‐expression networks 1–3 of the core genes differentially expressed in both lines expressing GH10 and both lines expressing GH11 xylanases in the wood‐forming tissues and their expression patterns in different tissues and in transgenic lines.
**Figure S9** Side co‐expression networks 4–6 of the core genes differentially expressed in both lines expressing GH10 and both lines expressing GH11 xylanases in the wood‐forming tissues and their expression patterns in different tissues and in transgenic lines.
**Figure S10** Side co‐expression network 7 of the core genes differentially expressed in both lines expressing GH10 and both lines expressing GH11 xylanases in the wood‐forming tissues and their expression patterns in different tissues and in transgenic lines.

**Table S1** Metabolomics data for wood‐forming tissues of transgenic lines expressing GH10 and GH11 xylanases compared to wild‐type plants.
**Table S2** Relative contents of metabolites identified in wood‐forming tissues of transgenic lines expressing GH10 and GH11 xylanases and wild‐type (WT) plants.
**Table S3** Relative contents of metabolites identified in wood‐forming tissues of transgenic lines expressing GH10 and GH11 xylanases and wild‐type plants (WT).
**Table S4** Gene expression data based on RNA sequencing in wood‐forming tissues of transgenic lines compared to WT for all genes significantly affected (Log_2_ > 0.5849625, *P*_adj_ < 0.01) in at least one line.
**Table S5** Genes significantly affected (Log_2_ > 0.5849625, *P*_adj_ <0.01) in both WP:GH10 lines (L10 and L17) and both WP:GH11 lines (L12 and L13).
**Table S6** GO enrichment for all genes significantly upregulated or downregulated in common in WP:GH10 and WP:GH11 lines (391 and 239 genes, respectively) based on Log_2_ > 0.5849625 and *P*_adj_ < 0.01.
**Table S7** Functional classification and co‐expression networks in the wood‐forming tissues of *P. tremula* of the genes differentially regulated in common in GH10‐ and GH11‐ expressing hybrid aspen lines.
**Table S8** Secondary cell wall‐related genes expressed in differentiating secondary wall‐forming tissues of aspen and their expression levels in transgenic aspen lines expressing fungal GH10 and GH11.
**Table S9** Genes specifically and significantly affected (Log_2_ > 0.5849625, *P*_adj_ < 0.01) in either both WP:GH10 lines (L10 and L17) or both WP:GH11 lines (L12 and L13).
**Table S10** GO enrichment for all genes significantly upregulated or downregulated either specifically in WP:GH10 lines 12 and 13 or in WP:GH11 lines 10 and 17 based on Log_2_ > 0.5849625 and *P*_adj_ < 0.01.
**Table S11** Primers used in this study.

## Data Availability

The raw RNA‐Seq data that support the findings of this study are available in the European Nucleotide Archive (ENA) at EMBL‐EBI (https://www.ebi.ac.uk/ena/browser/home), under accession no. PRJEB75802.
