# Supplementary material for: Modification of xylan in secondary walls alters cell wall biosynthesis and wood formation programs and improves saccharification
Source: Plant Biotechnol J. 2024 Oct 22;23(1):174–97. doi: 10.1111/pbi.14487 (PMC11672743; doi:10.1111/pbi.14487)
Supplement: Supplementary file 1 — Figure S1 Transgene expression levels in developing wood of transgenic lines expressing GH10 and GH11 xylanases based on RNA sequencing. Figure S2 Toluidine blue‐stained wood sections showing reduction in cell wall thickness and change in staining indicative of reduced lignin content in transgenic lines expressing xylanases. Figure S3 Fluorescence microscopy for detection of lignin in the wood tissue of transgenic lines expressing GH10 and GH11 xylanases. Figure S4 Oligomeric mass profiling (ESI‐MS) of acetylated glucuronoxylan extracted with 30 min subcritical water extraction from transgenic lines expressing GH10 and GH11 xylanases. Figure S5 Oligomeric mass profiling (ESI‐MS) of glucuronoxylan extracted with alkali from transgenic lines expressing GH10 and GH11 xylanases. Figure S6 Saccharification yields of mannose and galactose obtained from wood of transgenic lines expressing GH10 and GH11 xylanases. Figure S7 Main co‐expression network of the core genes differentially expressed in both lines expressing GH10 and both lines expressing GH11 xylanases in the wood‐forming tissues and their expression patterns in different tissues and transgenic lines. Figure S8 Side co‐expression networks 1–3 of the core genes differentially expressed in both lines expressing GH10 and both lines expressing GH11 xylanases in the wood‐forming tissues and their expression patterns in different tissues and in transgenic lines. Figure S9 Side co‐expression networks 4–6 of the core genes differentially expressed in both lines expressing GH10 and both lines expressing GH11 xylanases in the wood‐forming tissues and their expression patterns in different tissues and in transgenic lines. Figure S10 Side co‐expression network 7 of the core genes differentially expressed in both lines expressing GH10 and both lines expressing GH11 xylanases in the wood‐forming tissues and their expression patterns in different tissues and in transgenic lines. [file PBI-23-174-s001.docx]

14

*a*

*a*

*GH10*

*a*

*b*

*GH11*

*A A*

*c*

*d*

*B*

L20 L24 L11 L12 L13

L10 L17

35S:GH10

WP:GH10

WT WP:GH11 WT

12

10

VST expression

8

6

4

2

0

**Supplementary Figure S1. Transgene expression levels in developing wood of transgenic lines expressing GH10 and GH11 xylanases based on RNA sequencing**. Data are means ± SE, N=5 for transgenic lines and 8 for WT. Different letters above the bars indicate significant difference among averages (P≤0.05, Tukey test).

WT 35S:GH11 L8 35S:GH11 L9


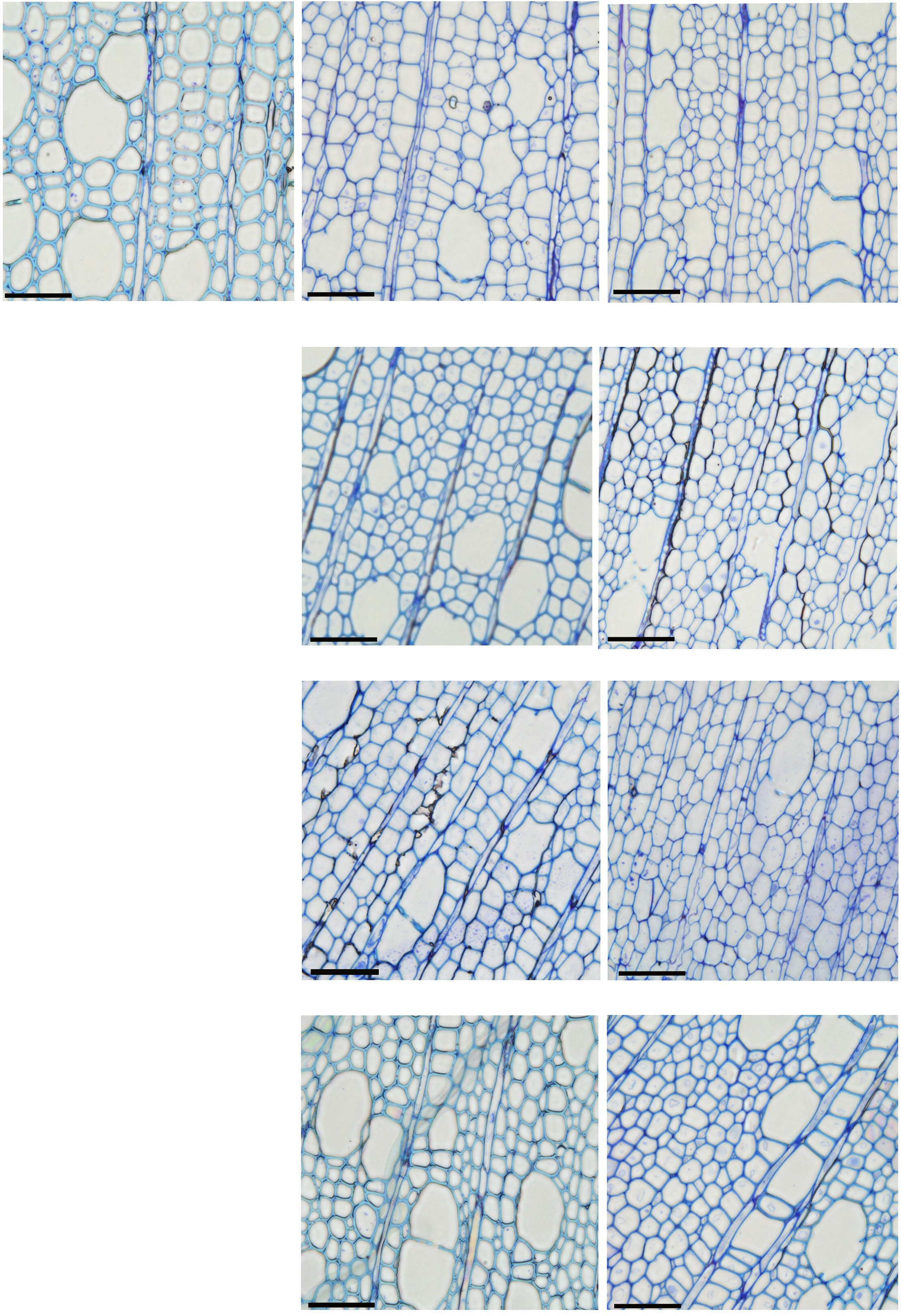


**Supplementary Figure S2. Toluidine blue stained wood sections showing reduction in cell wall thickness and change in staining indicative of reduced lignin content in transgenic lines expressing xylanases.** Some vessel elements in most affected lines show *irregular xylem phenotype* (*irx*). The phenotype is visible in all analyzed lines except WP:GH10_line 11, which had lower transgene expression than other lines. Scale bar = 50 µm

WP:GH11 L10 WP:GH11 L17

35S:GH10 L20 35S:GH10 L24

WP:GH10 L11 WP:GH10 L12


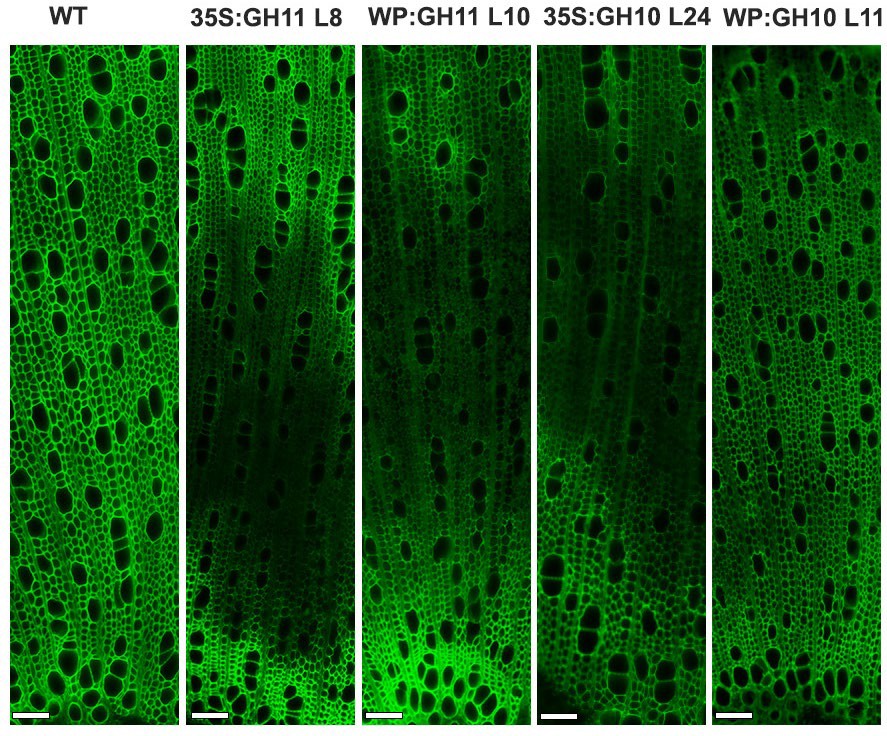


**Supplementary Figure S3. Fluorescence microscopy for detection of lignin in the wood tissue of transgenic lines expressing GH10 and GH11 xylanases.** Note the weak autofluorescence from the irregular xylem phenotypes of transgenic line. Scale bar= 50µm.


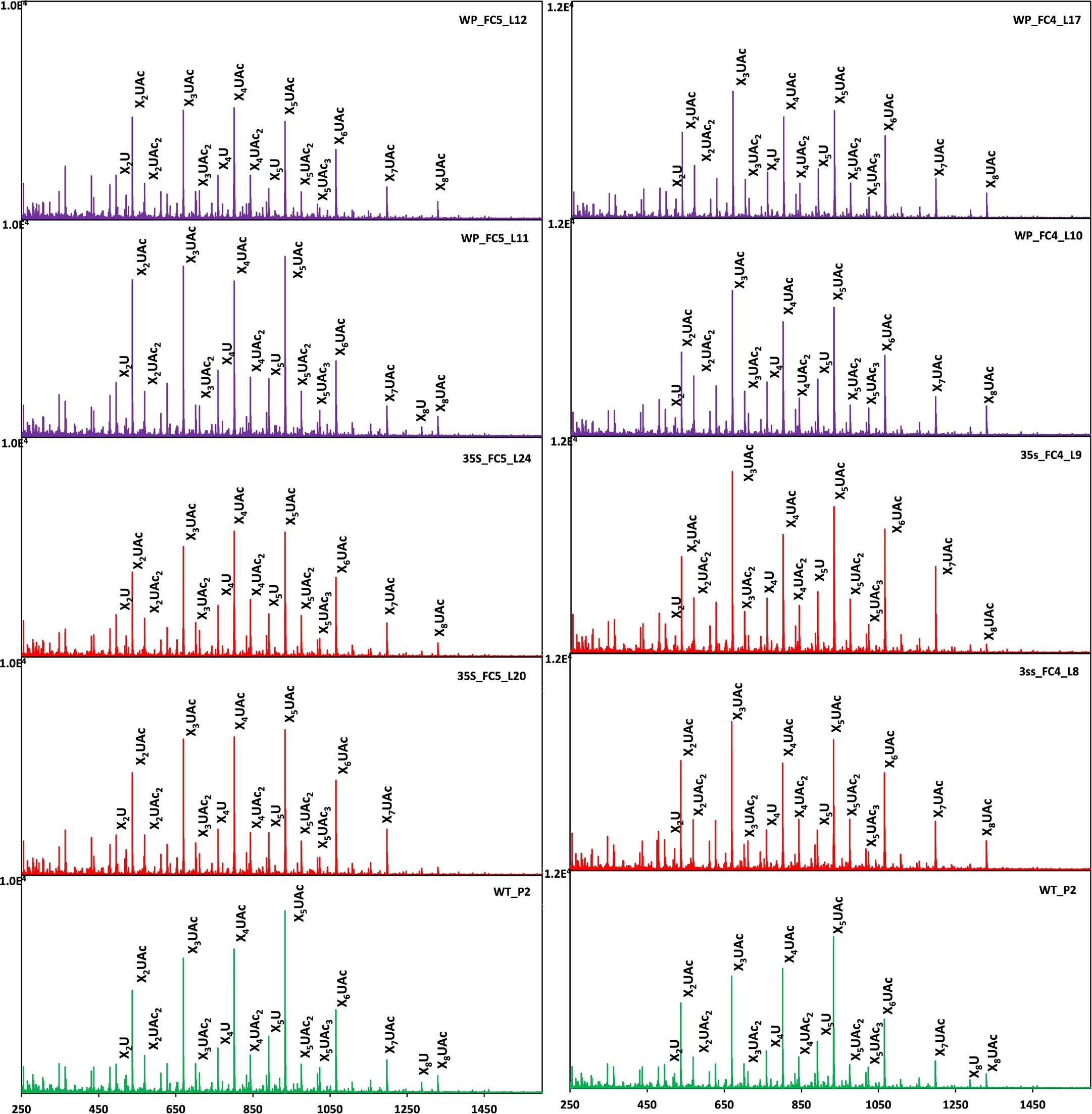
**WP:GH10 L12**

**WP:GH10 L11**

**WP:GH11 L17**

**WP:GH11 L10**

**35S:GH10 L24**

**ESI-MS counts**

**35S:GH10 L20**

**35S:GH11 L9**

**35S:GH11 8**

**ESI-MS counts**

**WT WT**

# *m/z m/z*

**Supplementary Figure S4. Oligomeric mass profiling (ESI-MS) of acetylated glucuronoxylan extracted with 30 min subcritical water extraction from transgenic lines expressing GH10 and GH11 xylanases.** The oligomers are released by incubating the extracted hemicellulose with GH30 glucuronoxylanase.

9E+02


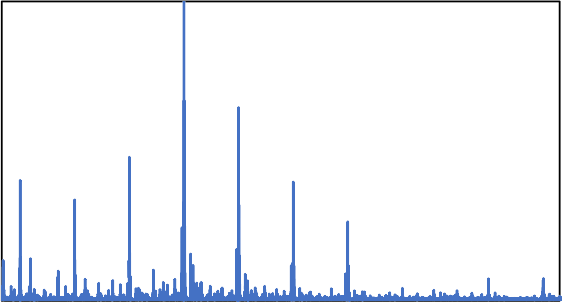

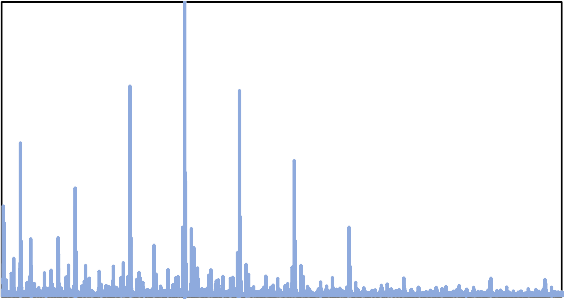

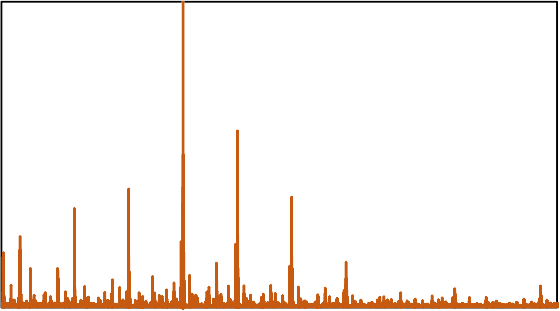

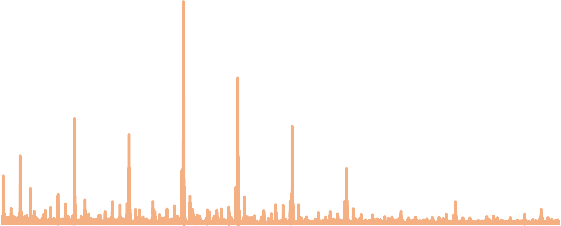


**35S:GH10 L24**

**WP:GH10 L11**

**WP:GH11 L17**

**WT**

**WT**

**35S:GH11 L8**

9E+02

**ESI-MS counts**

9E+02

**X5U**

**X6U**

**X5U**

**X6U**

0

**X2U**

**X3U**

**X4U**

**X7U**

**X8U**

**X10U**

**X2U**

**X3U**

**X4U**

**X7U**

**X8U**

**X10U**

450 950 1450

450 950 1450

# *m/z*

**Supplementary Figure S5. Oligomeric mass profiling (ESI-MS) of glucuronoxylan extracted with alkali from transgenic lines expressing GH10 and GH11 xylanases.** The oligomers are released by incubating the extracted hemicellullose with GH30 glucuronoxylanase.

0.020

120%

110%

0.015

Man yield (g g^-1^)

***

0.010

*

0.005

0.000

0.020

0.015

L20 L24 L11 L12 L8 L9 L10 L17

35S:GH10 WP:GH10 35S:GH11 WP:GH11 WT

L20 L24 L11 L12 L8 L9 L10 L17

35S:GH10 WP:GH10 35S:GH11 WP:GH11 WT

80%

Gal yield (g g^-1^)

120%

130%

140%

170%

150%

130%

0.010

40%

***

***

***

***

***

60%

0.005

***

***

*

***

0.000

110%

**Supplementary Figure S6. Saccharification yields of mannose and galactose obtained from wood of transgenic lines expressing GH10 and GH11 xylanases.** The sugars were released during acid pretreatment. Data are means ± SE, N = 3 or 6 technical replicates from the pooled material of 6 trees for transgenic lines and WT, respectively. * - P≤0.05; ** - P≤0.01; *** - P≤0.001 for comparisons with WT by Dunnett’s test.

*

**Supplementary Figure S7. Main co- expression network of the core genes differentially expressed in both lines expressing GH10 and both lines expressing GH11 xylanases in the wood-forming tissues and their expression patterns in different tissues and transgenic lines. (A)** Main co- expression network in the wood- forming tissues colored according to gene expression in wood developmental zones shown in the heatmap **(C). (B)-(D)** Heatmaps showing expression of the genes from the main expression network


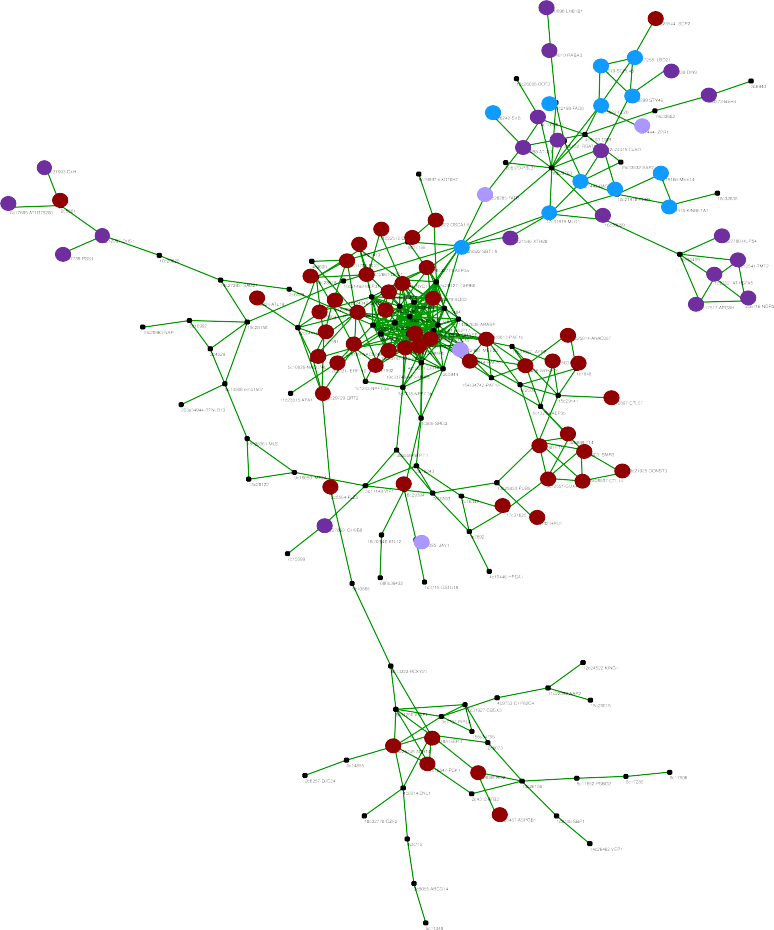


LHB1

B1

A

SCP2

RAB

A3

LBD

21

SCPL

45

DIN9

STY

46

BEH4

FAD8

FLZ6

SVB

Unk

ZPR1

RGA

T1

ATL

23

CuA

O

C4H

MEE

14

BASS

6

PSBY

FATB

Unk

Unk

MLO

1

KIN

BETA

1

Unk

OSC

A1.8

CP5

DHS

1

Unk

XTH

28

HUP

54

Unk

SBT

1.9

PSS1

RCI3

TMT

2

AEP3

a

Unk

DPD

1

HSFA

5

NYC1

DMP

ATL7

9

Unk

4 SQD

2

KMS GRIK

NDR

APG8 5

H

LLR3 1

1

Unk

HUP

17 LBD

ANA

C087

NAC

074a

MM CCD 19

PAF1

b

MM

Pb 1

Mee

23

NAC

074b ERF1

10

Pa

PAF1

c

ATL8

WRK Unk

3

Unk

Y75

QRT

2

CTL0

7

TT4

FT1

SMR

3

GON

ST3

GUX

4

unk

CTL1

4

FLZ6

Unk

HRU

1

GH9

B8

JAV1

GSR

1

ACH

T4

PCK

1

RD2

C

ASPG

B1

D

**(A)** in different tissues of aspen **(B),** wood developmental zones **(C),** and in transgenic lines (Log_2_ fold change compared to wild type) **(D).**

B

WP:GH11 L10 WP:GH11 L17 WP:GH10 L12 WP:GH10 L13

**PCD**

**SW**

**PW-SW**

**Ca**

**Ph**


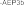

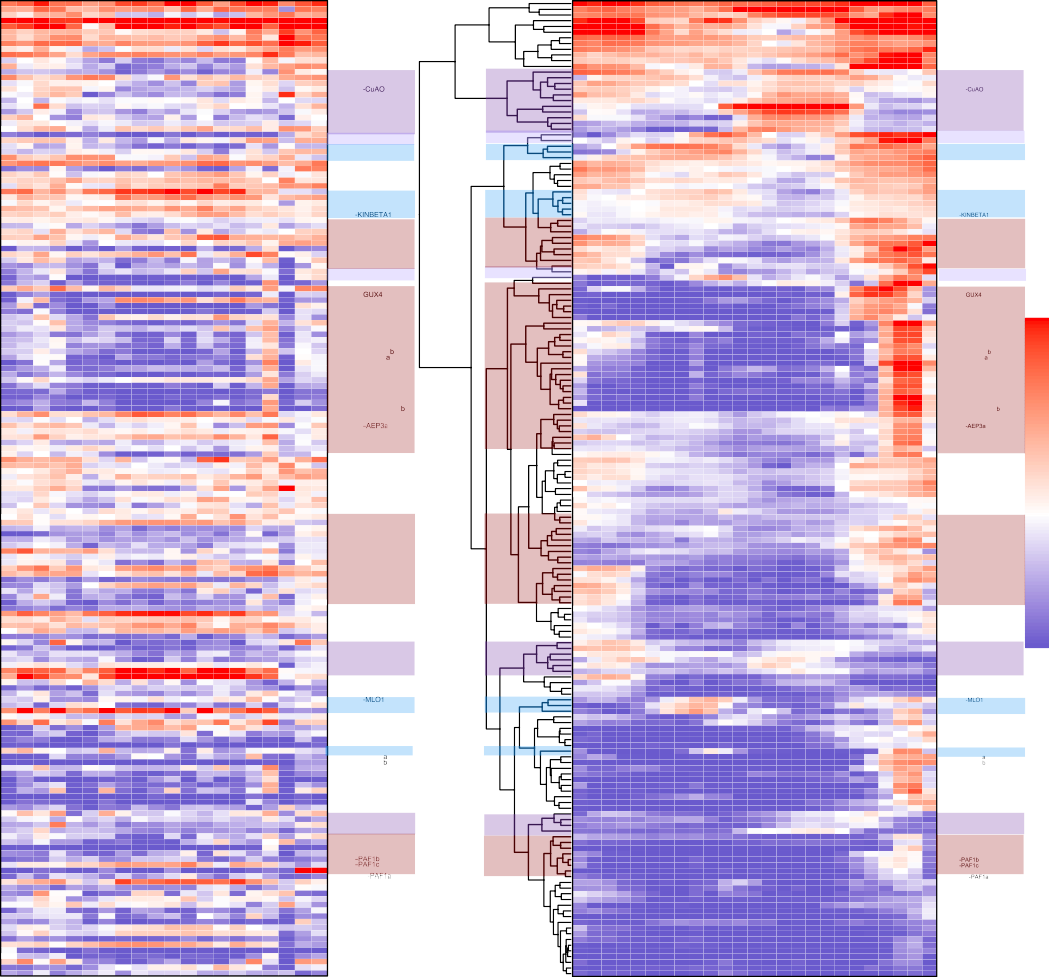

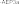

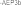

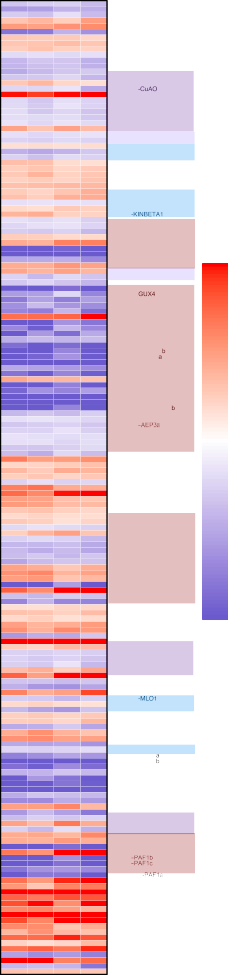


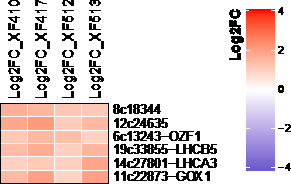
Side network 1


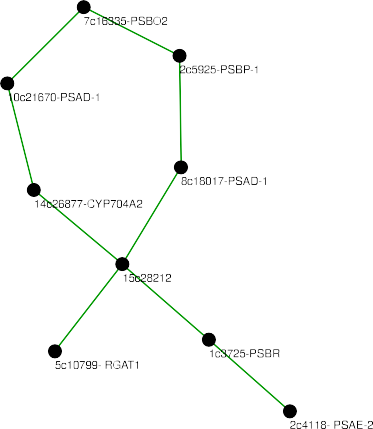

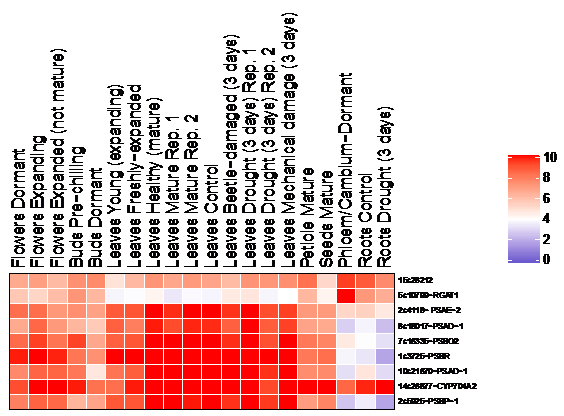

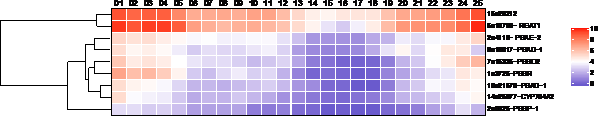

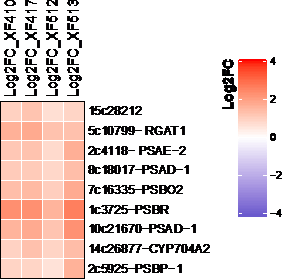


Side network 2

**Ph Ca PW-SW SW**

**PCD**


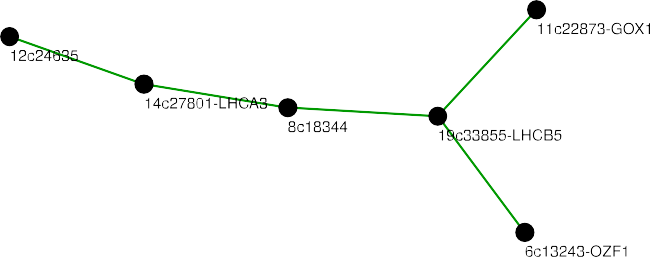

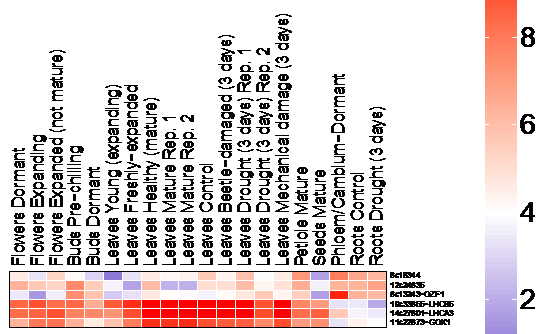

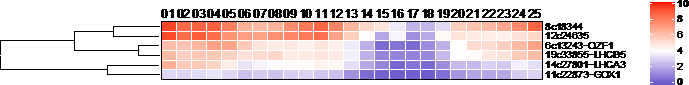


**Ph Ca PW-SW SW**

**PCD**

WP:GH11 L10 WP:GH11 L17 WP:GH10 L12 WP:GH10 L13

WP:GH11 L10 WP:GH11 L17 WP:GH10 L12 WP:GH10 L13

| **Ph** | **Ca** | **PW-SW** | **SW** | **PCD** |
| --- | --- | --- | --- | --- |

**Supplementary Figure S8. Side co-expression networks 1-3 of the core genes differentially expressed in both lines expressing GH10 and both lines expressing GH11 xylanases in the wood-forming tissues and their expression patterns in different tissues and in transgenic lines.** Note that network 3 includes genes downregulated or upregulated in the cambium.


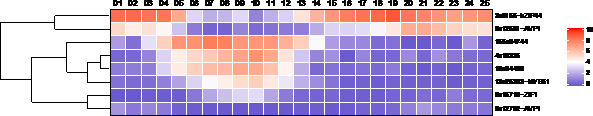

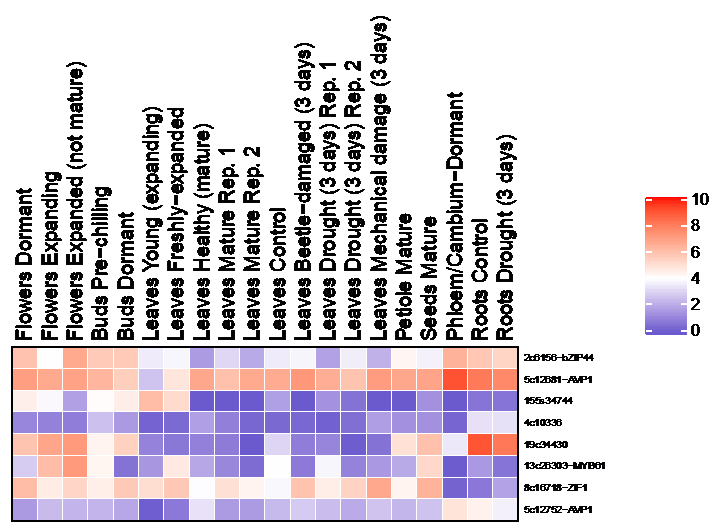

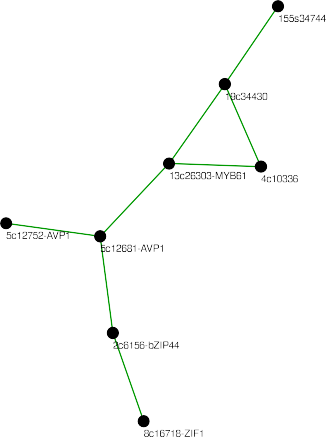


-SCREEM-like

Side network 3

-MLO4

-SCREEM-like

-MLO4

-SCREEM-l

-MLO4


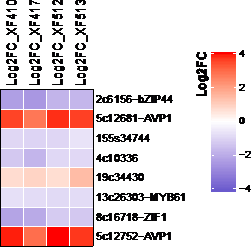


-SCREEM-like

-MLO4

WP:GH11 L10 WP:GH11 L17 WP:GH10 L12 WP:GH10 L13


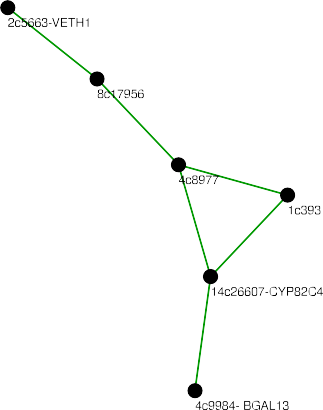

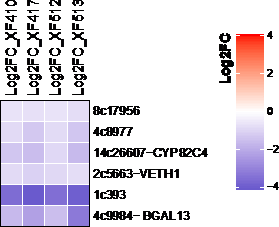

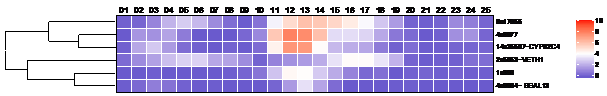

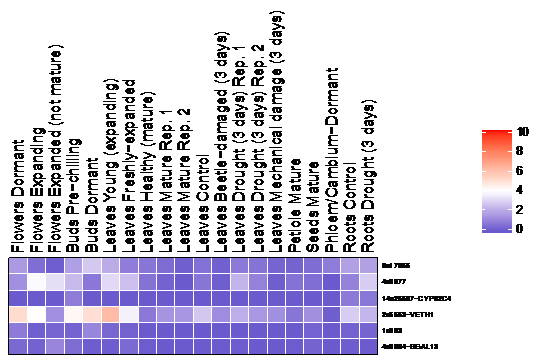


Side network 5

**Ph Ca PW-SW SW**

**PCD**


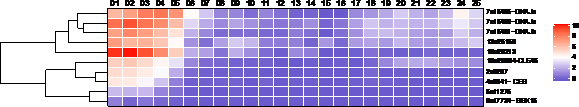


**Ph Ca PW-SW SW**

**PCD**

-TIM23


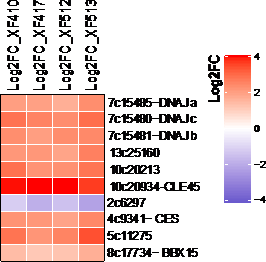


-TIM23

WP:GH11 L10 WP:GH11 L17 WP:GH10 L12 WP:GH10 L13

WP:GH11 L10 WP:GH11 L17 WP:GH10 L12 WP:GH10 L13

WP:GH11 L10 WP:GH11 L17 WP:GH10 L12 WP:GH10 L13


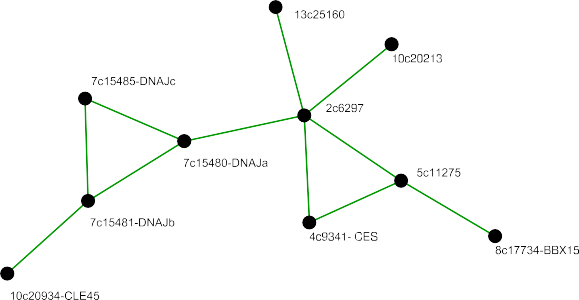

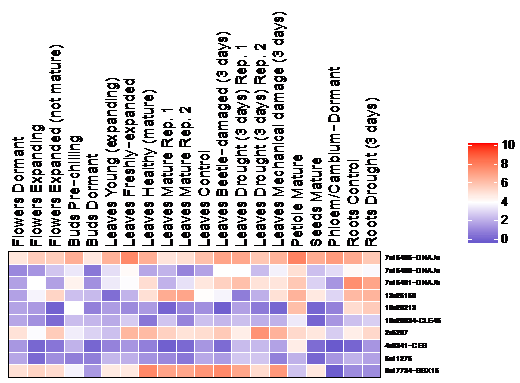


-TIM23

Side network 4

-TIM23

| **Ph** | **Ca** | **PW-SW** | **SW** | **PCD** |
| --- | --- | --- | --- | --- |


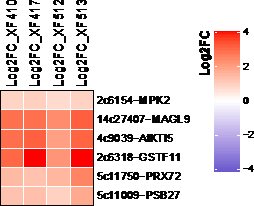
**Supplementary Figure S9. Side co-expression networks 4-6 of the core genes differentially expressed in both lines expressing GH10 and both lines expressing GH11 xylanases in the wood-forming tissues and their expression patterns in different tissues and in transgenic lines.** Note that networks 4 and 6 include genes specifically expressed in the phloem, which are mostly upregulated in transgenic lines, whereas network 5 includes genes upregulated during primary to secondary wall transition, which are downregulated in transgenic lines.


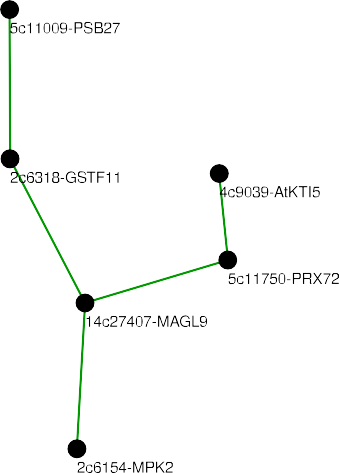

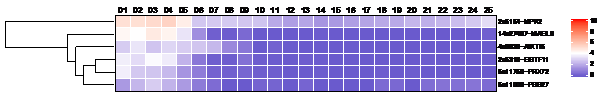

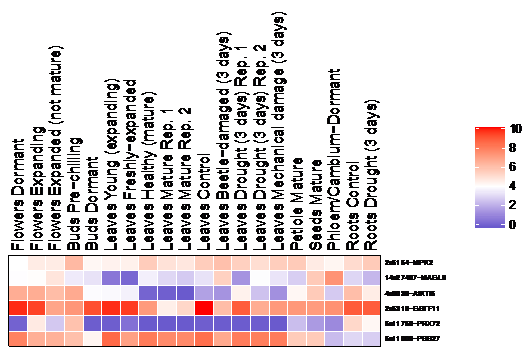


Side network 6


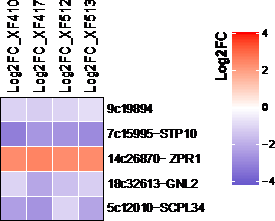


WP:GH11 L10 WP:GH11 L17 WP:GH10 L12 WP:GH10 L13

| **Ph** | **Ca** | **PW-SW** | **SW** | **PCD** |
| --- | --- | --- | --- | --- |


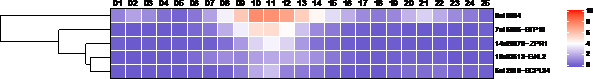


**Supplementary Figure S10. Side co-expression network 7 of the core genes differentially expressed in both lines expressing GH10 and both lines expressing GH11 xylanases in the wood-forming tissues and their expression patterns in different tissues and in transgenic lines.** Note that network 7 includes genes specifically upregulated during primary to secondary wall transition, which are downregulated in transgenic lines except for one, ZPR1.


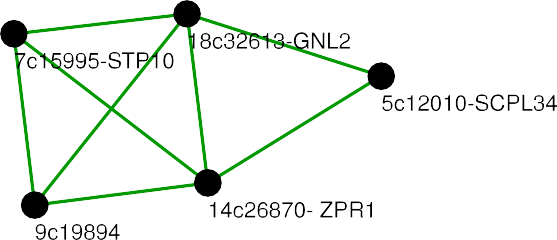

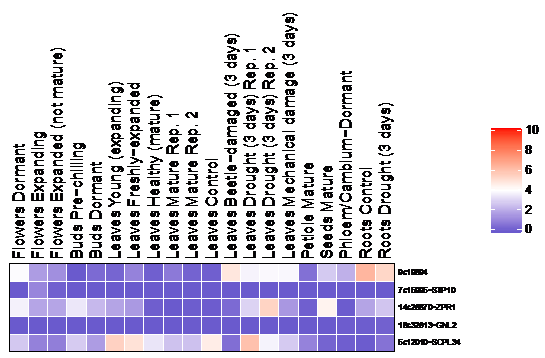


Side network 7
